# Infection-relevant conditions dictate differential versus coordinate expression of *Salmonella* chaperones and cochaperones

**DOI:** 10.1128/mbio.00227-25

**Published:** 2025-03-31

**Authors:** Carissa Chan, Keiichiro Mukai, Eduardo A. Groisman

**Affiliations:** 1 Department of Microbial Pathogenesis, Yale School of Medicine 198940 , New Haven, Connecticut, USA; The Ohio State University, Columbus, Ohio, USA

**Keywords:** DnaK, J-domain cochaperones, magnesium, PhoP, RpoH

## Abstract

Molecular chaperones are critical for protein homeostasis. In bacteria, chaperone trigger factor (TF) folds proteins co-translationally, and chaperone DnaK requires a J-domain cochaperone and nucleotide exchange factor GrpE to fold proteins largely post-translationally. However, when the pathogen *Salmonella enterica* serovar Typhimurium faces the infection-relevant condition of cytoplasmic Mg^2+^ starvation, DnaK reduces protein synthesis independently. This raises the possibility that bacteria differentially express chaperones and cochaperones. We now report that *S*. Typhimurium responds to cytoplasmic Mg^2+^ starvation by increasing mRNA amounts of *dnaK* while decreasing those of the TF-encoding gene *tig* and J-domain cochaperone genes *dnaJ* and *djlA*. This differential strategy requires the master regulator of Mg^2+^ homeostasis and virulence PhoP, which increases *dnaK* mRNA amounts by lowering the ATP concentration, thereby hindering proteolysis of the alternative sigma factor RpoH responsible for *dnaK* transcription. We also establish that DnaK exerts negative feedback on the RpoH protein and RpoH-dependent transcripts independently of J-domain cochaperones. Thus, bacteria express chaperones and cochaperones coordinately or differentially depending on the specific stress perturbing protein homeostasis.

**IMPORTANCE:**

Molecular chaperones typically require cochaperones to fold proteins and to prevent protein aggregation, and the corresponding genes are thus coordinately expressed. We have now identified an infection-relevant stress condition in which the genes specifying chaperone DnaK and cochaperone DnaJ are differentially expressed despite belonging to the same operon. This differential strategy requires the master regulator of Mg^2+^ homeostasis and virulence in the pathogen *Salmonella enterica* serovar Typhimurium. Moreover, it likely reflects that *Salmonella* requires *dnaK*, but not J-domain cochaperone-encoding genes, for survival against cytoplasmic Mg^2+^ starvation and expresses genes only when needed. Thus, the specific condition impacting protein homeostasis determines the coordinate versus differential expression of molecular chaperones and cochaperones.

## INTRODUCTION

Proteins carry out the vast majority of cellular processes and must be folded correctly to function. Folding takes place both during and after protein synthesis (1), and both intrinsically (2, 3) and with molecular chaperones that help protein clients adopt their proper conformations (4). Molecular chaperones also play critical roles under stress conditions, such as heat shock and oxidative stress, that cause protein misfolding and aggregation (5, 6). Some chaperones operate alone, and others require additional factors, such as cochaperones and nucleotide exchange factors (7). In addition, a given chaperone may require additional factors to operate under one protein homeostasis-disrupting condition but function without such factors under a different condition (8).

**Fig 1:**
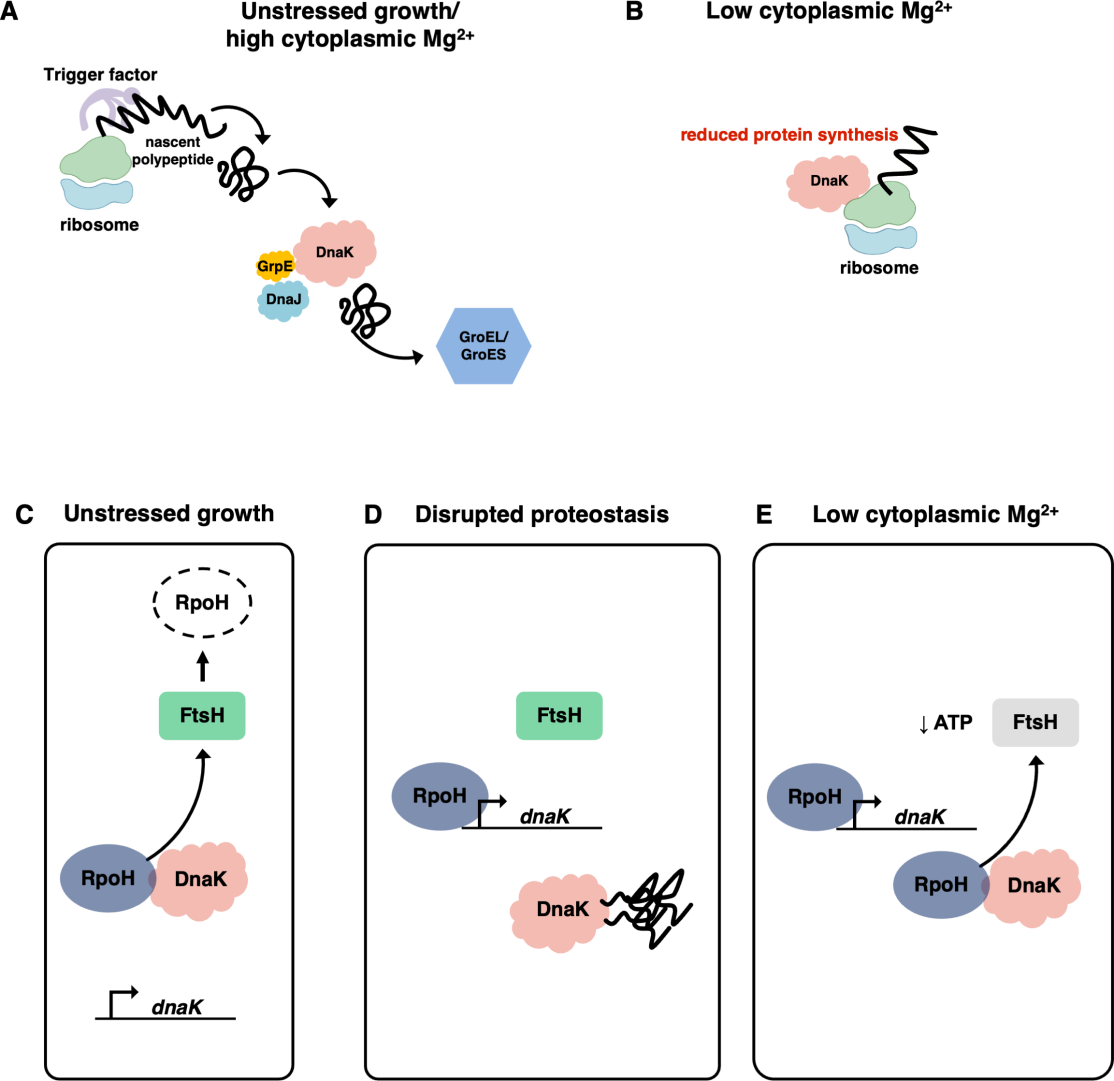
Chaperones fold bacterial proteins during unstressed growth and in *Salmonella* experiencing infection-relevant stress. (A) During unstressed growth, bacterial proteins are folded by a chaperone network consisting of the ribosome-associated chaperone trigger factor, the DnaK/DnaJ/GrpE chaperone system, and the GroEL/GroES chaperone system. (B) During cytoplasmic Mg^2+^ starvation, DnaK binds ribosomes and reduces protein synthesis independently of J-domain cochaperones. (C) During unstressed growth, DnaK delivers the alternative sigma factor RpoH for degradation by the FtsH protease, reducing the amount of RpoH available to promote transcription of the *dnaK* gene. (D) Under conditions that disrupt proteostasis, DnaK is titrated by aggregated proteins, thus liberating RpoH from being delivered to FtsH for proteolysis and increasing the amount of RpoH available to promote transcription of the *dnaK* gene. (E) During cytoplasmic Mg^2+^ starvation, the ATP concentration decreases, which reduces the activity of the ATP-dependent FtsH protease, thereby increasing the amount of RpoH available to promote transcription of the *dnaK* gene.

Bacteria use three major chaperone systems: (i) the ribosome-associated bacteria-specific trigger factor (TF), which folds proteins co-translationally as they emerge from the ribosome (9); (ii) the DnaK/DnaJ/GrpE system, consisting of chaperone DnaK, J-domain cochaperone DnaJ, and nucleotide exchange factor GrpE (10), which acts largely post-translationally (11, 12) and maintains proteome quality by aiding protein folding and preventing protein aggregation (13); and (iii) the essential GroEL/GroES system, consisting of chaperone GroEL and cochaperone GroES, which acts solely post-translationally (14) (Fig. 1A).

*Escherichia coli* responds to heat shock by increasing transcription of the *dnaKdnaJ* and *groESgroEL* operons from promoters dependent on the alternative sigma factor RpoH (also referred to as sigma 32 or σ32) (5) but does not alter transcription of the TF-encoding *tig* gene (15). Bacteria control RpoH abundance at multiple levels, including transcription initiation (16), translation (17, 18) (Fig. 1B), stability (19, 20) (Fig. 1C), and activity (21–23). The DnaK/DnaJ/GrpE system plays a critical role in the magnitude and duration of the heat shock response by exerting negative feedback on RpoH (22, 24–26). Tight control of RpoH abundance avoids the growth inhibition resulting from excess RpoH (27).

The pathogen *Salmonella enterica* serovar Typhimurium faces cytoplasmic Mg^2+^ starvation during infection (28), a stress that imperils protein homeostasis (29). This is because *S*. Typhimurium responds to cytoplasmic Mg^2+^ starvation by decreasing the ATP concentration (30, 31), likely due to ~85% of the cellular ATP existing as a Mg^2+^ salt (32, 33). The decrease in ATP concentration hinders protein solubility (34) and reduces protein synthesis (35) and regulated proteolysis by ATP-dependent proteases (36), including FtsH, which is responsible for RpoH degradation (37–39). Cytoplasmic Mg^2+^ starvation hyperactivates PhoP (40, 41), the master regulator of Mg^2+^ homeostasis and virulence (42) that governs the reduction in ATP concentration (31, 35, 36). In addition, it decreases TF binding to ribosomes ~30-fold while increasing DnaK binding to ribosomes threefold (8). DnaK binding to ribosomes reduces protein synthesis and confers survival against hyperosmotic stress and cytoplasmic Mg^2+^ starvation independently of *tig* and the three J-domain cochaperone genes *cbpA*, *djlA*, and *dnaJ* (8).

The contrasting requirements for chaperones and cochaperones in bacteria facing cytoplasmic Mg^2+^ starvation versus other protein homeostasis-perturbing conditions led us to investigate whether the corresponding genes are differentially expressed. Here, we report that cytoplasmic Mg^2+^ starvation upregulates expression of the *dnaK* gene while downregulating expression of the *dnaJ*, *djlA*, and *tig* genes. This differential strategy is governed by PhoP, which promotes transcription from RpoH-dependent promoters by hindering RpoH proteolysis. We also establish that DnaK’s C-terminal domain is essential for negative feedback on the abundance of RpoH and RpoH-activated transcripts, whereas the J-domain cochaperones are dispensable. Our results reveal that the specific stress threatening protein homeostasis determines the differential versus coordinate expression of chaperones and cochaperones.

## RESULTS

### Cytoplasmic Mg^2+^ starvation increases *dnaK* mRNA amounts, but not *dnaJ*’s, in a *phoP*-dependent manner

The *dnaK* and *dnaJ* genes are believed to form an operon (43). We explored the possibility of these genes being differentially expressed during cytoplasmic Mg^2+^ starvation because DnaK displays DnaJ-independent activities under this stress condition (8) and also because 85 nt separate the stop codon of the upstream *dnaK* gene from the start codon of the downstream *dnaJ* gene, which is an unusually long distance for bacterial genes that are part of the same transcription unit (44). Thus, we examined *dnaK* and *dnaJ* mRNA amounts in wild-type *S*. Typhimurium grown in defined media with different Mg^2+^ concentrations and for different extents of time. These conditions were designed to reveal expression behaviors resulting from low cytoplasmic Mg^2+^, low extracytoplasmic Mg^2+^, and a Mg^2+^-abundant environment.

**Fig 2:**
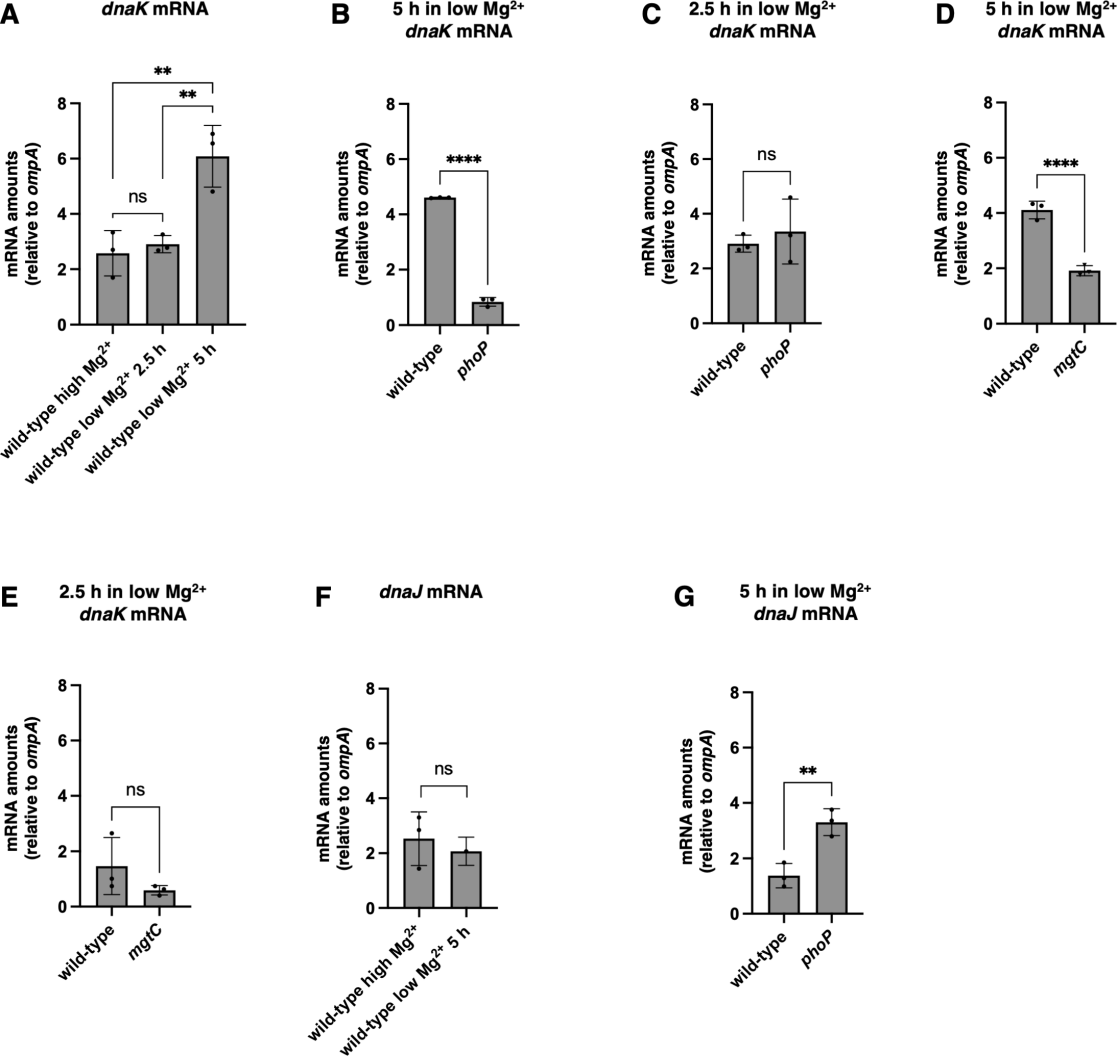
Low cytoplasmic Mg^2+^ increases expression of *dnaK*, but not *dnaJ*, in a PhoP-dependent manner. (A) mRNA abundance of the *dnaK* gene relative to that of the constitutive *ompA* control in wild-type (14028s) *S*. Typhimurium following 4.5 h of growth in high (10 mM) Mg^2+^, 2.5 h of growth in low (10 µM) Mg^2+^, or 5 h of growth in low (10 µM) Mg^2+^. (B) mRNA abundance of the *dnaK* gene relative to that of the constitutive *ompA* control in wild-type (14028s) and *phoP* (MS7953s) *S*. Typhimurium following 5 h of growth in low (10 µM) Mg^2+^. (C) mRNA abundance of the *dnaK* gene relative to that of the constitutive *ompA* control in wild-type (14028s) and *phoP* (MS7953s) *S*. Typhimurium following 2.5 h of growth in low (10 µM) Mg^2+^. (D) mRNA abundance of the *dnaK* gene relative to that of the constitutive *ompA* control in wild-type (14028s) and *mgtC* (EL1) *S*. Typhimurium following 5 h of growth in low (10 µM) Mg^2+^. (E) mRNA abundance of the *dnaK* gene relative to that of the constitutive *ompA* control in wild-type (14028s) and *mgtC* (EL1) *S*. Typhimurium following 2.5 h of growth in low (10 µM) Mg^2+^. (F) mRNA abundance of the *dnaJ* gene relative to that of the constitutive *ompA* control in wild-type (14028s) *S*. Typhimurium following 4.5 h of growth in high (10 mM) Mg^2+^ or 5 h of growth in low (10 µM) Mg^2+^. (G) mRNA abundance of the *dnaJ* gene relative to that of the constitutive *ompA* control in wild-type (14028s) and *phoP* (MS7953s) *S*. Typhimurium following 5 h of growth in low (10 µM) Mg^2+^. Statistical analysis was performed using two-tailed Student’s *t-*test comparing the indicated sample group to the wild-type sample group or comparing the bracketed sample groups (ns = not significant). Data in panels A–G represent mean ± SD of three independent biological replicates. Statistical analysis was performed using two-tailed Student’s *t*-test comparing the bracketed sample groups (**P < 0.01, ****P < 0.0001, ns = not significant).

The mRNA amounts of the *dnaK* gene were threefold higher in bacteria grown in 10 µM Mg^2+^ for 5 h, a time when *S*. Typhimurium experiences cytoplasmic Mg^2+^ starvation (31, 35, 45), than when grown in Mg^2+^-abundant (10 mM Mg^2+^) conditions (Fig. 2A). The higher *dnaK* mRNA amounts present in bacteria grown in 10 µM Mg^2+^ for 5 h result from low cytoplasmic Mg^2+^ rather than low extracytoplasmic Mg^2+^ because *dnaK* mRNA amounts were similar in bacteria grown in 10 mM Mg^2+^ for 4.5 h and in 10 µM Mg^2+^ for 2.5 h (Fig. 2A), which is prior to the onset of cytoplasmic Mg^2+^ starvation (35).

We reasoned that PhoP is responsible for the higher *dnaK* mRNA amounts in low cytoplasmic Mg^2+^ because it controls the expression of several genes critical for protein homeostasis activated during cytoplasmic Mg^2+^ starvation (42, 46). A *phoP* null mutant had threefold less *dnaK* mRNA than the wild-type strain following growth in 10 µM Mg^2+^ for 5 h (Fig. 2B). The effect of the *phoP* mutation is specific to low cytoplasmic Mg^2+^ because *dnaK* mRNA amounts were similar in the two strains following 2.5 h in 10 µM Mg^2+^ (Fig. 2C), a condition in which PhoP controls the response to low extracytoplasmic Mg^2+^, such as chemical modification of the bacterial cell surface (42).

The PhoP-activated *mgtC* gene encodes a protein that exerts positive feedback on PhoP by limiting PhoP proteolysis by the ATP-dependent protease ClpAP and its adaptor ClpS (41). We determined that the *mgtC* mutant had twofold less *dnaK* mRNA than wild-type *S*. Typhimurium following growth in 10 µM Mg^2+^ for 5 h (Fig. 2D). By contrast, wild-type and *mgtC* strains had similar *dnaK* mRNA amounts following growth in 10 µM Mg^2+^ for 2.5 h (Fig. 2E), the low extracytoplasmic Mg^2+^ condition that does not promote transcription of the *mgtC* coding region (47).

The increased *dnaK* expression resulting from cytoplasmic Mg^2+^ starvation resembles that exhibited by other genes promoting cytoplasmic protein homeostasis (42). By contrast, *dnaJ* mRNA amounts were similar following growth in low Mg^2+^ for 5 h or high Mg^2+^ for 4.5 h (Fig. 2F), and the *phoP* mutant had higher *dnaJ* mRNA amounts than the wild-type strain following growth in 10 µM Mg^2+^ for 5 h (Fig. 2G). These results indicate that the *dnaK* and *dnaJ* genes are differentially expressed in bacteria facing cytoplasmic Mg^2+^ limitation despite being similarly expressed under other conditions (43).

### Cytoplasmic Mg^2+^ starvation does not promote expression of J-domain cochaperone genes

*S*. Typhimurium specifies three J-domain cochaperones: CbpA, DjlA, and DnaJ (48). We considered the possibility of cytoplasmic Mg^2+^ starvation promoting expression of the *cbpA* and/or *djlA* genes to make up for *dnaJ* mRNA abundance not increasing alongside *dnaK*’s following growth in low Mg^2+^ for 5 h (Fig. 2A and F). However, *cbpA* mRNA amounts were similar following growth in low or high Mg^2+^ (Fig. S1A) in wild-type and *phoP* mutant strains (Fig. S1B). Curiously, *djlA* mRNA amounts were more than fourfold lower following growth in low Mg^2+^ compared to high Mg^2+^ (Fig. S1C) and twofold higher in the *phoP* mutant than in the wild-type strain when grown in low Mg^2+^ (Fig. S1D). These results indicate that cytoplasmic Mg^2+^ starvation decreases *djlA* expression in a PhoP-dependent manner, the opposite of what happens with *dnaK* (Fig. 2B).

Together with the data presented in the previous section, these results establish that cytoplasmic Mg^2+^ starvation promotes expression of *dnaK* but not of genes encoding its three canonical J-domain cochaperones. The selective increase in *dnaK* mRNA may reflect that DnaK reduces protein synthesis and confers survival independently of all three J-domain cochaperones under cytoplasmic Mg^2+^ starvation (8).

### The DnaK to DnaJ protein ratio increases during cytoplasmic Mg^2+^ starvation

**Fig 3:**
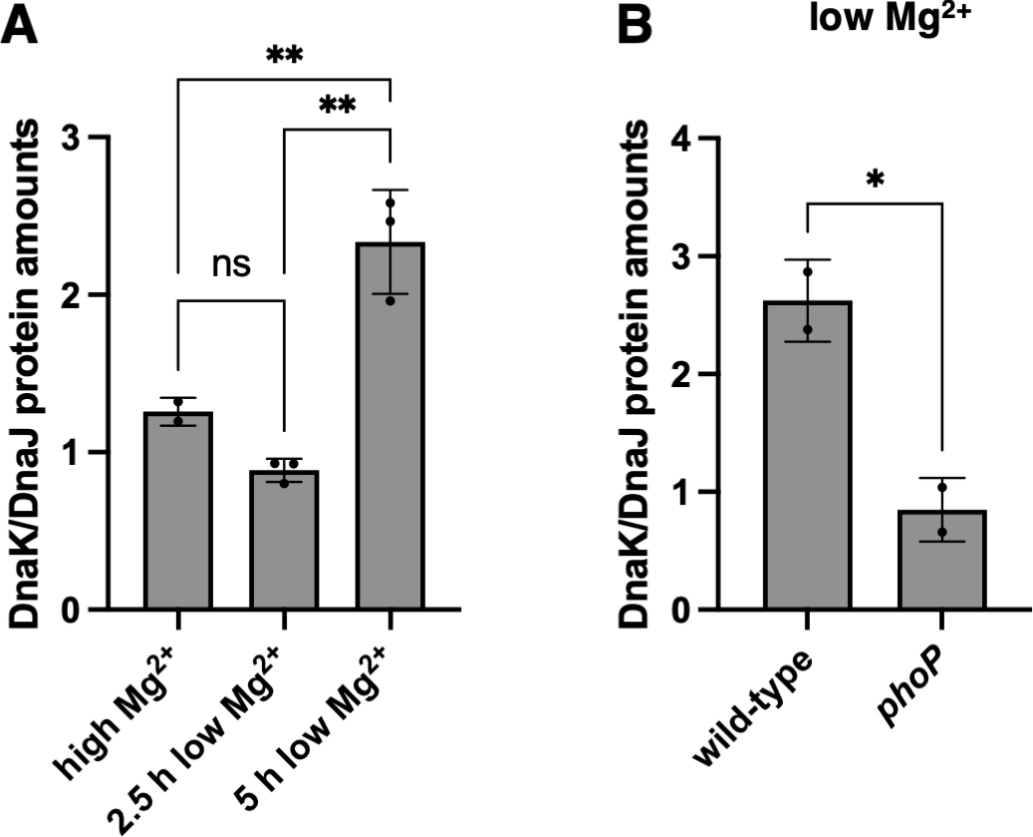
Low cytoplasmic Mg^2+^ increases the DnaK:DnaJ protein ratio in a PhoP-dependent manner. (A) Ratio of DnaK protein to DnaJ protein determined by Western blot in wild-type (14028s) *S*. Typhimurium following 4.5 h of growth in high (10 mM) Mg^2+^, 2.5 h of growth in low (10 µM) Mg^2+^, or 5 h of growth in low (10 µM) Mg^2+^. (B) Ratio of DnaK protein to DnaJ protein determined by Western blot in wild-type (14028s) and *phoP* (MS7953s) *S*. Typhimurium following 5 h of growth in low (10 µM) Mg^2+^. Statistical analysis was performed using two-tailed Student’s *t-*test comparing the indicated sample group to the wild-type sample group or comparing the bracketed sample groups (ns = not significant). Data in panels A and B represent mean ± SD of three independent biological replicates. Statistical analysis was performed using two-tailed Student’s *t-*test comparing the bracketed sample groups (*P < 0.05, **P < 0.01, ns = not significant).

The DnaK to DnaJ protein ratio was twofold higher during cytoplasmic Mg^2+^ starvation than in Mg^2+^-abundant conditions (Fig. 3A), largely reflecting the mRNA amounts of the corresponding genes (Fig. 2A and F). By contrast, bacteria grown in 10 µM Mg^2+^ for 2.5 h had the same DnaK to DnaJ protein ratio as those experiencing Mg^2+^-abundant conditions (Fig. 3A). The DnaK to DnaJ protein ratio was lower in the *phoP* mutant than in the wild-type strain during cytoplasmic Mg^2+^ starvation (Fig. 3B), echoing PhoP’s effect on the mRNA amounts of the corresponding genes (Fig. 2B and G). Thus, cytoplasmic Mg^2+^ starvation increases the DnaK to DnaJ ratio.

### Cytoplasmic Mg^2+^ starvation reduces *tig* expression in a PhoP-dependent manner

**Fig 4:**
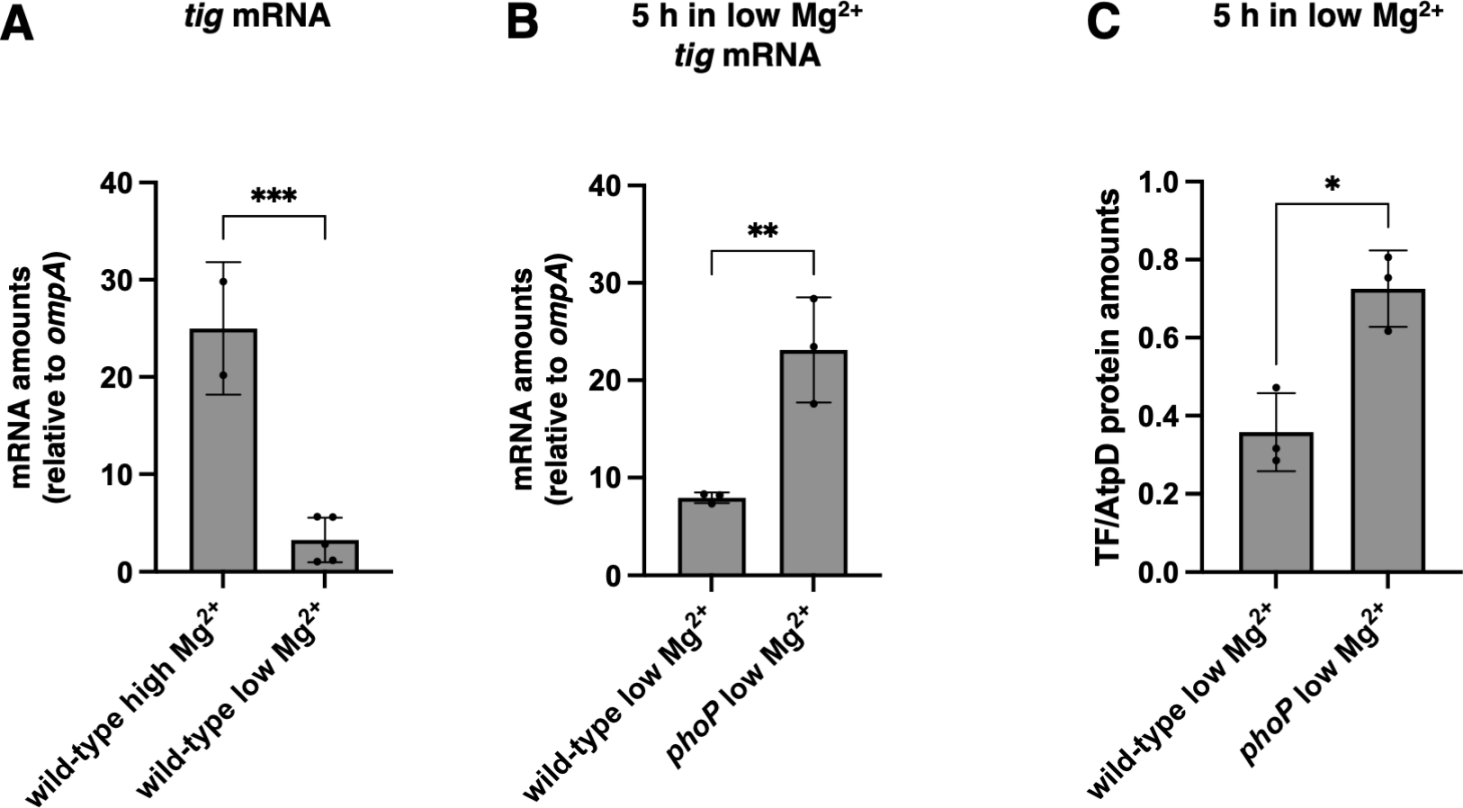
Low cytoplasmic Mg^2+^ reduces expression of trigger factor in a PhoP-dependent manner. (A) mRNA abundance of the *tig* gene relative to that of the constitutive *ompA* control in wild-type (14028s) *S*. Typhimurium following 4.5 h of growth in high (10 mM) Mg^2+^ or 5 h of growth in low (10 µM) Mg^2+^. (B) mRNA abundance of the *tig* gene relative to that of the constitutive *ompA* control in wild-type (14028s) and *phoP* (MS7953s) *S*. Typhimurium following 5 h of growth in low (10 µM) Mg^2+^. (C) Protein amounts of TF relative to the AtpD loading control determined by Western blot in wild-type (14028s) and *phoP* (MS7953s) *S*. Typhimurium following 5 h of growth in low (10 µM) Mg^2+^. Data in panels A–C represent mean ± SD of three independent biological replicates. Statistical analysis was performed using two-tailed Student’s *t-*test comparing the bracketed sample groups (*P < 0.05, **P < 0.01, ***P < 0.001, ns = not significant).

The amount of *tig* mRNA was eightfold lower in wild-type *S*. Typhimurium experiencing cytoplasmic Mg^2+^ starvation than under Mg^2+^-abundant conditions (Fig. 4A). The *phoP* mutant had threefold higher *tig* mRNA amounts (Fig. 4B) and twofold higher TF protein amounts (Fig. 4C) than the wild-type strain following growth in 10 µM Mg^2+^ for 5 h. However, TF protein amounts were similar during growth in 10 mM Mg^2+^ and 10 µM Mg^2+^ in the wild-type strain (Fig. S2). These results suggest that TF protein stabilization counters the reduction in *tig* mRNA amounts taking place during cytoplasmic Mg^2+^ starvation (Fig. 4A), possibly due to decreased proteolysis by ATP-dependent proteases (36).

### PhoP destabilizes the *dnaJ* mRNA during cytoplasmic Mg^2+^ starvation

**Fig 5:**
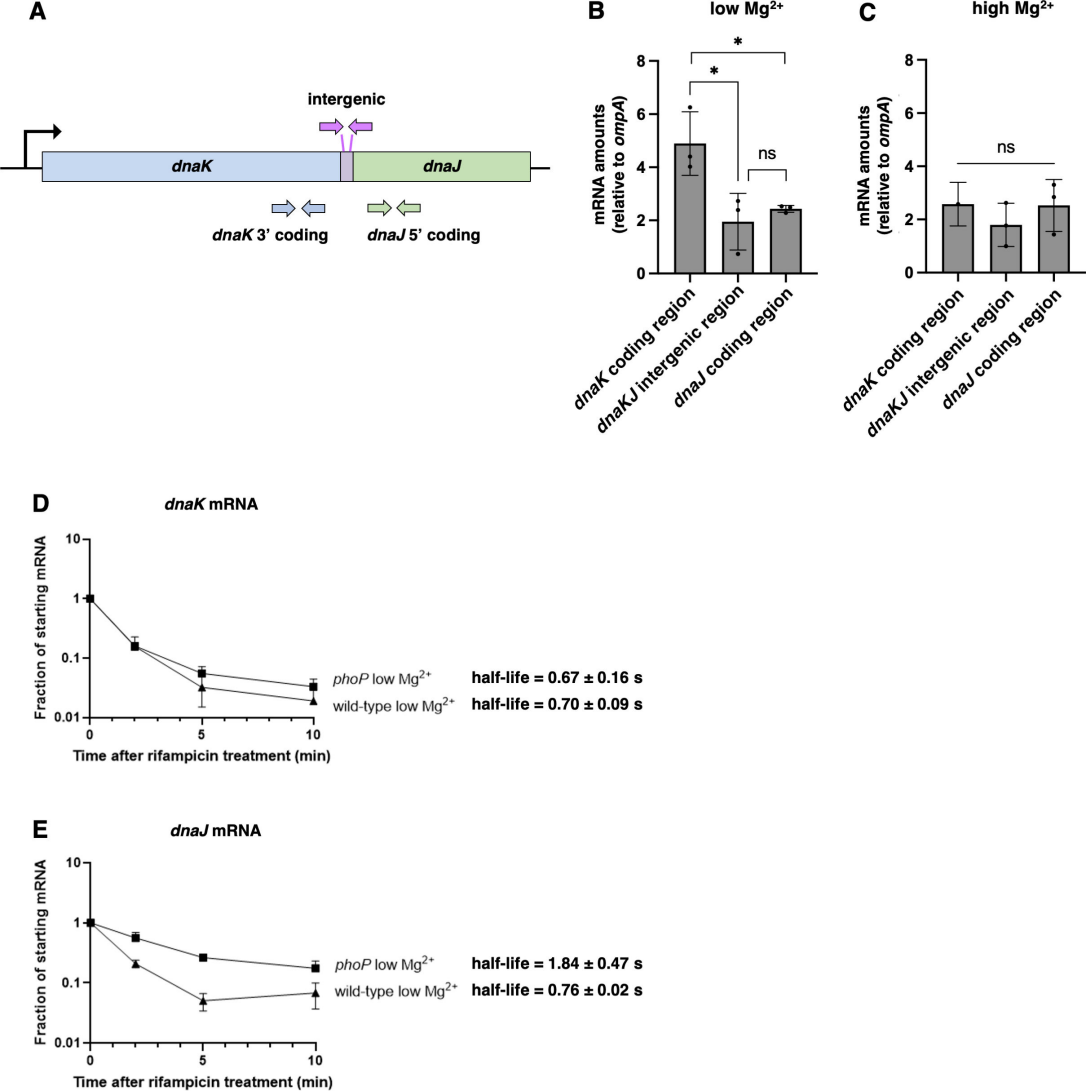
The *dnaK* and *dnaJ* genes are separately regulated during low cytoplasmic Mg^2+^. (A) Schematic of the *dnaKdnaJ* operon and intergenic region indicating the location of primers used to examine different parts of the transcript. Primers correspond to nucleotides 1797 to 1900 of the 1,917 nt long *dnaK* gene, −48 to +49 of the 85 nt long intergenic region, and 31 to 102 of the 1,140 nt long *dnaJ* gene. (B) mRNA abundance of the *dnaK* coding region, *dnaKJ* intergenic region, and *dnaJ* coding region relative to the constitutive *ompA* control in wild-type (14028s) *S*. Typhimurium following 5 h of growth in low (10 µM) Mg^2+^. (C) mRNA abundance of the *dnaK* coding region, *dnaKJ* intergenic region, and *dnaJ* coding region relative to the constitutive *ompA* control in wild-type (14028s) *S*. Typhimurium following 4.5 h of growth in high (10 mM) Mg^2+^. (D) *In vivo* stability of *dnaK* mRNA upon stopping new RNA synthesis using the RNA synthesis inhibitor rifamycin (200 µg/mL) in wild-type (14028s) and *phoP* (MS7953s) *S*. Typhimurium strains following growth in low (10 µM) Mg^2+^ for 5 h. Please note the logarithmic scale of the *y*-axis. (E) *In vivo* stability of *dnaJ* mRNA upon stopping new RNA synthesis using the RNA synthesis inhibitor rifamycin (200 µg/mL) in wild-type (14028s) and *phoP* (MS7953s) *S*. Typhimurium following 5 h of growth in low (10 µM) Mg^2+^. Please note the logarithmic scale of the *y*-axis. Data in panels B–E represent mean ± SD of three independent biological replicates. Statistical analysis was performed using two-tailed Student’s *t*-test comparing the bracketed sample groups (*P < 0.05, ns = not significant).

To explore how cytoplasmic Mg^2+^ starvation differentially alters expression of the *dnaK* and *dnaJ* genes, we examined the mRNA abundance of the *dnaK* and *dnaJ* coding regions and of the 85 nt-long *dnaK-dnaJ* intergenic region (Fig. 5A). In bacteria experiencing cytoplasmic Mg^2+^ starvation, the mRNA abundance of the *dnaK* coding region was ~2.5-fold higher than that corresponding to the *dnaK-dnaJ* intergenic region or the *dnaJ* coding region (Fig. 5B). By contrast, the mRNA amounts of the three regions were similar when bacteria were grown in high Mg^2+^ (Fig. 5C).

We explored the possibility of PhoP regulating the mRNA stabilities of the *dnaK* and *dnaJ* coding regions by growing wild-type and *phoP* strains in low (10 µM) Mg^2+^ for 5 h, halting new transcription by adding the RNA polymerase inhibitor rifampicin, and measuring the mRNA abundance of the *dnaK* and *dnaJ* coding regions over time. The mRNA stability of the *dnaK* coding region was similar in isogenic wild-type and *phoP* strains (Fig. 5D). By contrast, the half-life of the *dnaJ* coding region mRNA was 2.5-fold lower in the wild-type strain than in the *phoP* mutant (Fig. 5E). Together with the data presented above, these results suggest that PhoP increases *dnaK* transcription but decreases *dnaJ* mRNA stability.

### PhoP increases *dnaK* mRNA abundance by stabilizing the RpoH protein

**Fig 6:**
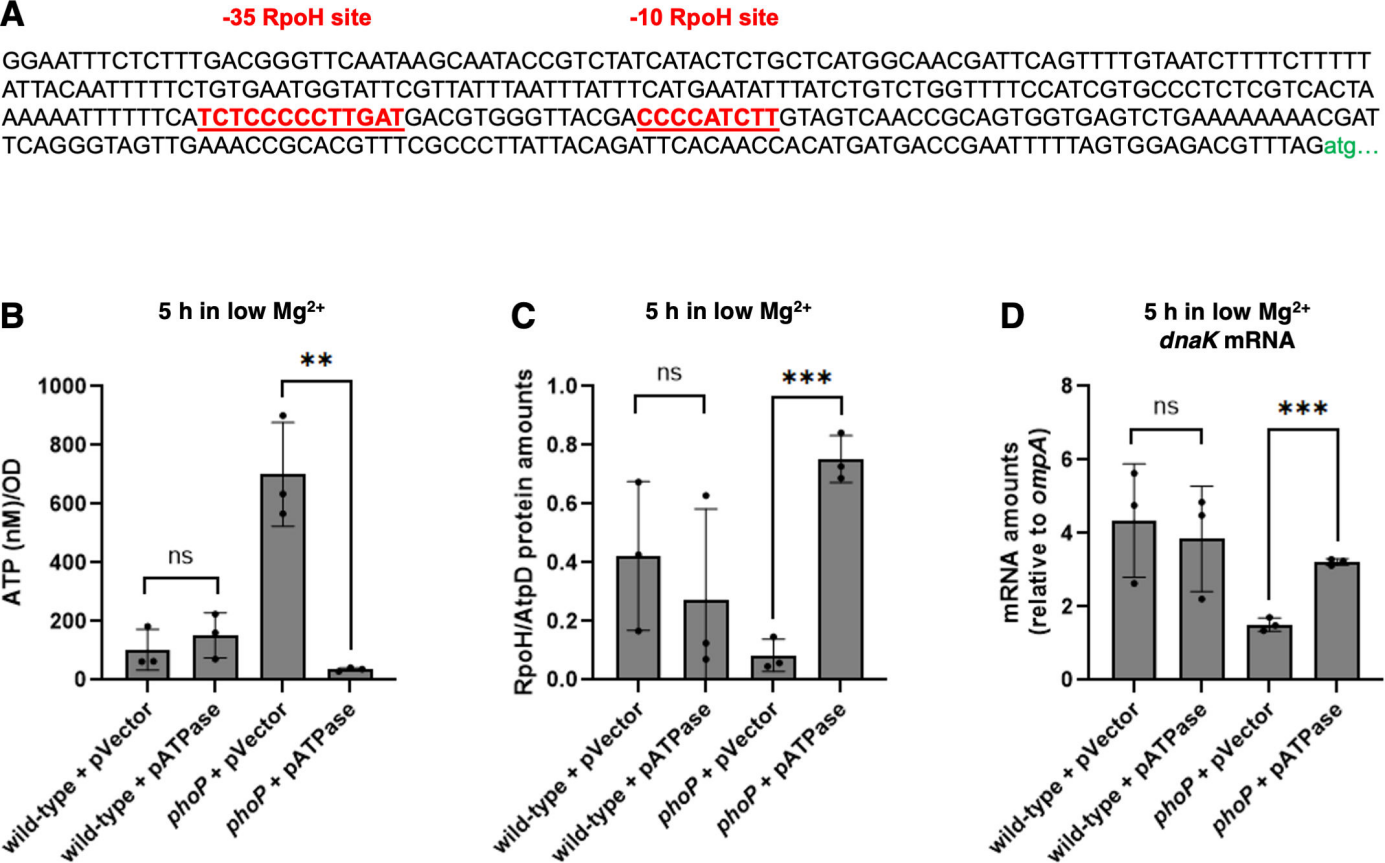
A reduction in ATP amounts increases RpoH abundance and expression of *dnaK*. (A) Nucleotide sequence of the *S*. Typhimurium *dnaK* promoter region harboring putative RpoH binding sites shown in red color. The *dnaK* start codon is in lower case green color. (B) ATP amounts in wild-type (14028s) and *phoP* (MS7953s) *S*. Typhimurium harboring the vector control or the AtpAGD-expressing plasmid following 5 h of growth in low (10 µM) Mg^2+^. (C) Protein amounts of RpoH relative to the AtpD loading control determined by Western blot in wild-type (14028s) and *phoP* (MS7953s) *S*. Typhimurium harboring the vector control or the AtpAGD-expressing plasmid following 5 h of growth in low (10 µM) Mg^2+^. (D) mRNA abundance of the *dnaK* gene relative to that of the constitutive *ompA* control in wild-type (14028s) and *phoP* (MS7953s) *S*. Typhimurium harboring the vector control or the AtpAGD-expressing plasmid following 5 h of growth in low (10 µM) Mg^2+^. Data in panels B–D represent mean ± SD of three independent biological replicates. Statistical analysis was performed using two-tailed Student’s *t-*test comparing the bracketed sample groups (**P < 0.01, ***P < 0.001, ns = not significant).

To understand how PhoP promotes *dnaK* transcription, we first looked for PhoP binding sites (49) in the 347 nt that separate *dnaK*’s start codon from the upstream *yaaI* gene but found none (Fig. 6A). By contrast, we identified two putative RpoH binding sites that are 100% conserved with those present in the *dnaK* promoter region from *E. coli* (Fig. 6A), activated by RpoH (50). The consensus sequences for RpoH-regulated promoters of *E. coli* are TNtCNCcCTTGAA at −35 and CCCCATtTa at −10, where lowercase letters indicate a tendency toward, but not an absolute requirement for, a certain nucleotide at that location (Biocyc.org). Thus, PhoP appears to promote *dnaK* transcription via RpoH.

We explored the possibility of PhoP activating *rpoH* transcription but found no PhoP binding sites (49) in the *rpoH* promoter region and similar *rpoH* mRNA amounts in wild-type and *phoP* strains following bacterial growth in low (10 µM) Mg^2+^ for 5 h (Fig. S3). These results suggested that PhoP increases RpoH abundance and/or activity indirectly. For example, PhoP governs the reduction in ATP amounts taking place during cytoplasmic Mg^2+^ starvation (31), which stabilizes RpoH(36).

To determine whether PhoP promotes *dnaK* transcription by stabilizing RpoH, we examined the amounts of RpoH protein and *dnaK* mRNA in wild-type and *phoP* strains harboring the AtpAGD-expressing plasmid or the vector control. The AtpAGD-expressing plasmid harbors the *atpA*, *atpG*, and *atpD* genes, which specify the proteins that make up the soluble subunit of the F_1_F_0_ ATP synthase (AtpAGD), the expression of which decreases ATP amounts (51), thereby limiting proteolysis of RpoH(36).

The AtpAGD-expressing plasmid corrected the abnormally high ATP amounts of the *phoP* mutant (Fig. 6B) and increased the amounts of both RpoH protein (Fig. 6C) and *dnaK* mRNA (Fig. 6D). That is, ATP amounts were sevenfold higher in the *phoP* mutant than in the wild-type strain, both carrying the plasmid vector (Fig. 6B). The *phoP* mutant with the AtpAGD-expressing plasmid had lower ATP amounts (Fig. 6B) and higher RpoH amounts (Fig. 6C) than the wild-type strain with either plasmid (Fig. 6B and C). Curiously, the *phoP* mutant carrying the AtpAGD-expressing plasmid had similar *dnaK* mRNA amounts as the wild-type strain with either plasmid (Fig. 6D).

The results presented above establish that PhoP increases *dnaK* mRNA amount by stabilizing RpoH. Moreover, they suggest that PhoP increases *dnaK* mRNA amounts by an additional mechanism.

### A C-terminal truncation of DnaK increases RpoH amounts and activity, but removal of the three J-domain cochaperones does not

The DnaK/DnaJ/GrpE system is reported to control the amount and activity of RpoH in *E. coli* (22, 24–26). During unstressed growth, the DnaK/DnaJ/GrpE system keeps RpoH in low abundance by promoting RpoH degradation. During heat shock, RpoH abundance and activity are proposed to increase because this stress promotes protein unfolding, and unfolded proteins titrate DnaK, rendering it unavailable to promote RpoH degradation and to inhibit RpoH activity (24, 52, 53).

We investigated the possibility of DnaK exerting negative feedback on RpoH without the participation of DnaJ or other J-domain cochaperones in *S*. Typhimurium because DnaK reduces protein synthesis and confers survival against cytoplasmic Mg^2+^ starvation independently of cochaperones in this bacterial species (8). Thus, we compared the amounts of RpoH protein and RpoH-activated transcripts in isogenic wild-type, *dnaK14* single mutant, and *cbpA djlA dnaJ* triple mutant *S*. Typhimurium strains. The *dnaK14* mutant specifies a stable version of the DnaK protein lacking its C-terminal 74 amino acids (8) and unable to reduce protein synthesis (8). The *dnaK14* mutant grows like the wild-type strain in both complex media and defined media with high (10 mM) Mg^2+^ but is defective for survival at 24 h in defined media with low (10 µM) Mg^2+^ (8).

**Fig 7:**
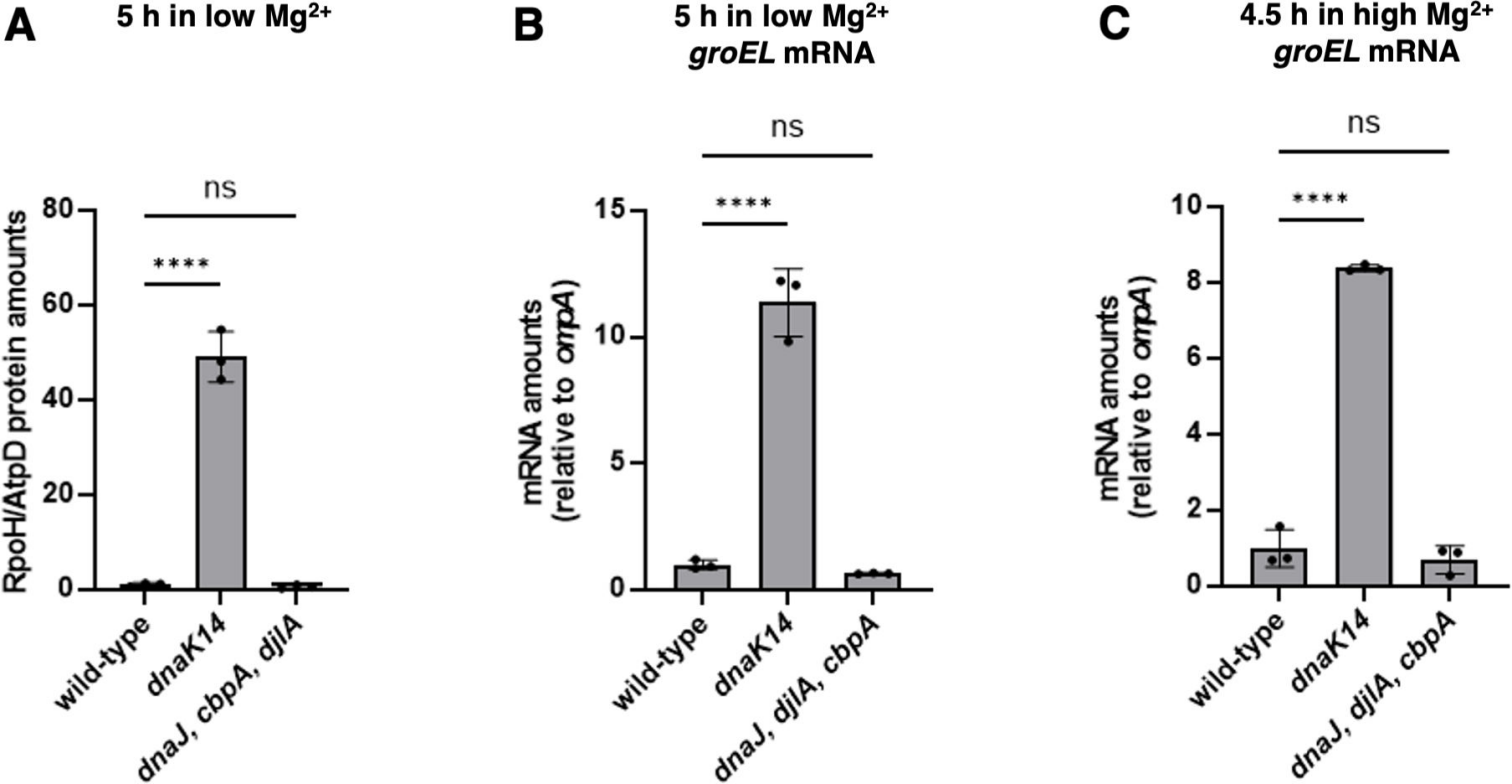
DnaK downregulates RpoH amounts and activity even in the absence of J-domain cochaperones. (A) Protein amounts of RpoH relative to the AtpD loading control determined by Western blot in wild-type (14028s), *dnaK14* (CC186), and *dnaJ cbpA djlA* (CC656) *S*. Typhimurium following 5 h of growth in low (10 µM) Mg^2+^. (B) mRNA abundance of the *groEL* gene relative to that of the constitutive *ompA* control in wild-type (14028s), *dnaK14* (CC186), and *dnaJ cbpA djlA* (CC656) *S*. Typhimurium following 5 h of growth in low (10 µM) Mg^2+^. (C) mRNA abundance of the *groEL* gene relative to that of the constitutive *ompA* control in wild-type (14028s), *dnaK14* (CC186), and *dnaJ cbpA djlA* (CC656) *S*. Typhimurium following 4.5 h of growth in high (10 mM) Mg^2+^. Data in panels A–C represent mean ± SD of three independent biological replicates. Statistical analysis was performed using two-tailed Student’s *t*-test comparing the bracketed sample groups (****P < 0.0001, ns = not significant).

RpoH amounts were 50-fold higher in the *dnaK14* mutant than in the wild-type strain or the *cbpA djlA dnaJ* triple mutant following bacterial growth in low (10 µM) Mg^2+^ for 5 h (Fig. 7A), a condition in which the *dnaK14* mutant exhibits wild-type survival (Fig. S4A). By contrast, the *cbpA djlA dnaJ* triple mutant had slightly lower (twofold less) RpoH amounts than the wild-type strain (Fig. 7A). Thus, the C-terminal domain of DnaK decreases RpoH abundance in a J-domain cochaperone-independent manner.

The mRNA amounts of the RpoH-activated *groEL* gene were 12 times higher in the *dnaK14* mutant than in the wild-type strain (Fig. 7B). The mRNA amounts of the RpoH-activated *creA* gene were also higher in the *dnaK14* mutant than the wild-type strain (Fig. S4C), but the difference was smaller than that observed with the *groEL* mRNA (Fig. 7B). Curiously, the mRNA amounts of the *groEL* and *creA* mRNAs were ~50% lower in the *cbpA djlA dnaJ* triple mutant than in the wild-type strain (Fig. 7B; Fig. S4C). These results indicate that the DnaK-mediated decrease in RpoH amounts reduces the mRNA abundance of RpoH-activated genes. Moreover, they suggest that J-domain cochaperones may hinder DnaK’s ability to reduce RpoH amounts and activity.

The negative feedback that the C-terminal domain of DnaK exerts on the RpoH protein and its regulated genes was also observed when bacteria were grown in high (10 mM) Mg^2+^ for 4.5 h (Fig. 7C; Fig. S4B and D) indicating that it is not exclusive to cytoplasmic Mg^2+^ starvation. Our results obtained with *S*. Typhimurium further support the notion that DnaK negatively regulates its transcriptional activator RpoH, as originally reported for *E. coli* (22, 24–26).

### The *dnaK* and *dnaJ* genes are not always syntenic

Genes encoding products that participate in the same biochemical and/or physiological pathways are often adjacent to one another. Such gene relationship, known as synteny, is believed to aid gene survival upon horizontal gene transfer (54, 55). This is because a foreign DNA segment is more likely to readily benefit an organism if it includes all the genes participating in a given pathway. Otherwise, the acquired foreign DNA will likely mutate and eventually be lost.

The *dnaK* and *dnaJ* genes belong to the same operon in many species (43), likely because DnaK operates with DnaJ and nucleotide exchange factor GrpE when performing its canonical protein folding role (56). However, *dnaK* and *dnaJ* are differentially expressed during cytoplasmic Mg^2+^ starvation (Fig. 2), and DnaK reduces protein synthesis in a J-domain cochaperone-independent manner (8), indicating that DnaK can operate independently of DnaJ and suggesting that *dnaK* and *dnaJ* may not be syntenic in other bacterial species.

**Fig 8:**
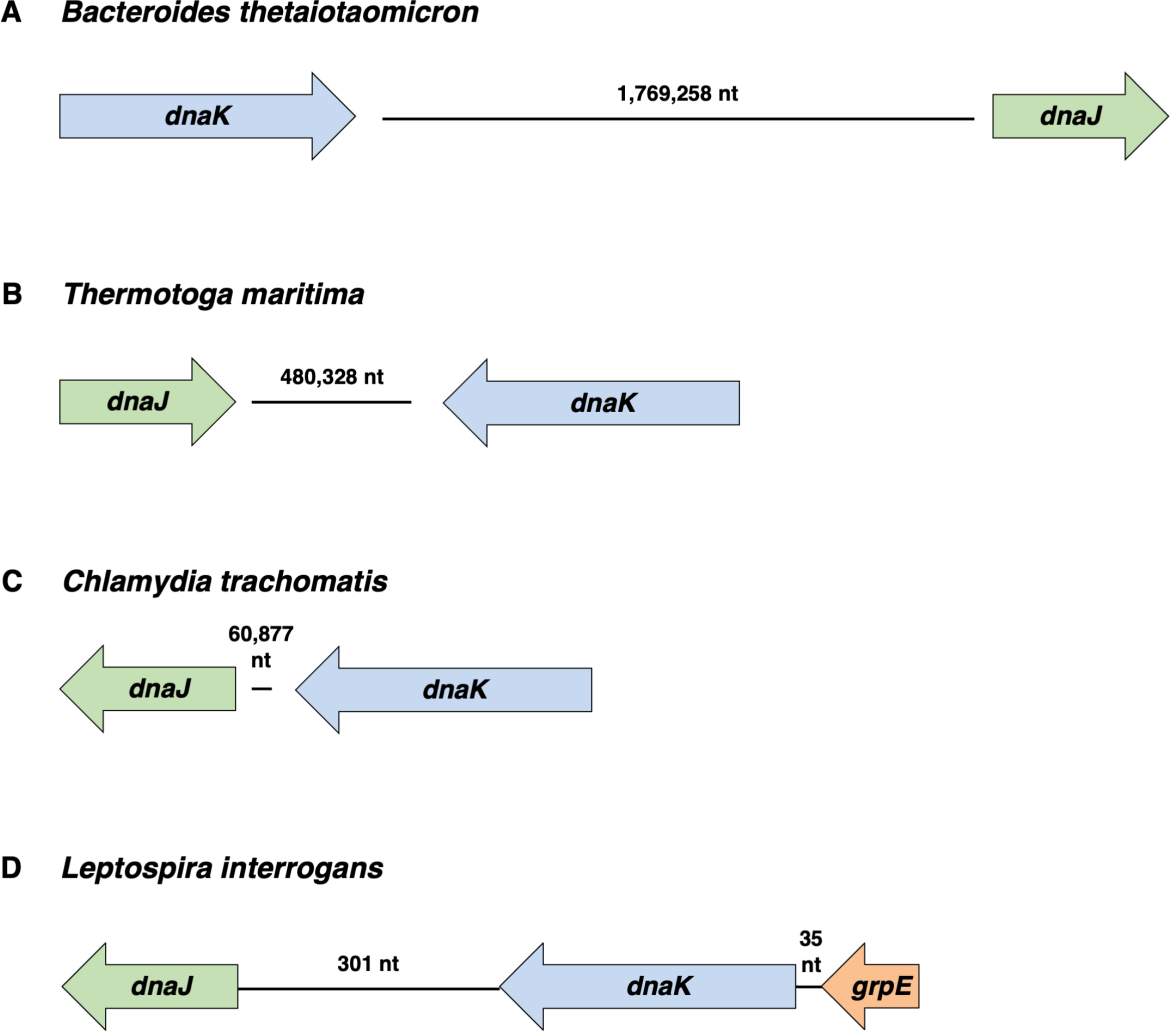
The organization of the *dnaK* and *dnaJ* genes varies among the genomes of bacterial species. (A) Organization of the *dnaK* and *dnaJ* genes in the genome of *Bacteroides thetaiotaomicron* strain VPI-5482. (B) Organization of the *dnaK* and *dnaJ* genes in the genome of *Thermotoga maritima* strain MSB8. (C) Organization of the *dnaK* and *dnaJ* genes in the genome of *Chlamydia trachomatis* strain L2. (D) Organization of the *dnaK*, *dnaJ*, and *grpE* genes in the genome of *Leptospira interrogans* strain 22.

The genome of the gut commensal bacterium *Bacteroides thetaiotaomicron* strain VPI-5482 harbors an open reading frame (*BT4615*) that shares 61% amino acid identity (75% similarity) with the *S*. Typhimurium DnaK protein and another ORF (*BT1244*) that shares 43% amino acid identity (59% similarity) with the *S*. Typhimurium DnaJ protein, 25% identity (43% similarity) with the longer *S*. Typhimurium CbpA protein, and no significant identity with the *S*. Typhimurium DjlA protein. Curiously, the *BT4615* and *BT1244* genes are 1,769,258 nt apart in the circular *B. thetaiotaomicron* genome (Fig. 8A). Encoding a *dnaJ* sequelog, the *BT1244* gene is located immediately adjacent to and in the same orientation as the *BT1243* gene, which specifies a sequelog of nucleotide exchange factor GrpE. The *dnaK* and *dnaJ* genes are not syntenic in the genomes of the thermophilic bacterium *Thermotoga maritima* strain MSB8 (Fig. 8B) or the obligate intracellular bacterium *Chlamydia trachomatis* (Fig. 8C) either.

## DISCUSSION

Molecular chaperones play diverse roles in protein homeostasis and exhibit distinct requirements for cochaperones. The widely distributed chaperone DnaK requires a J-domain cochaperone to refold proteins when *E. coli* experiences heat shock (10), but it reduces protein synthesis without J-domain cochaperones in *S*. Typhimurium facing cytoplasmic Mg^2+^ starvation (8). We have now established that (i) DnaK and its J-domain cochaperones are regulated in opposite ways when the proteostasis-perturbing stress is cytoplasmic Mg^2+^ starvation (Fig. 2A and F); (ii) PhoP, the master regulator of Mg^2+^ homeostasis (42), promotes *dnaK* transcription (Fig. 2B) but destabilizes the *dnaJ* mRNA (Fig. 5E) even though *dnaK* and *dnaJ* are part of a polycistronic mRNA under other growth conditions (Fig. 2A) (57); (iii) PhoP furthers *dnaK* transcription by stabilizing RpoH (Fig. 6C), the alternative sigma factor directly responsible for transcription from the *dnaK* promoter in *E. coli* (50); and (iv) DnaK requires its C-terminal domain but no J-domain cochaperones to decrease RpoH amount and activity (Fig. 7), reinforcing the notion that DnaK can operate independently of J-domain cochaperones (8). Because bacteria express their genes only when needed, the coordinate versus differential expression of chaperone DnaK and its J-domain cochaperones reflects the specific proteostasis-perturbing stress faced by bacteria.

Cells experiencing stresses that compromise protein homeostasis typically respond by upregulating pathways involved in protein maintenance and repair (1). J-domain cochaperones participate in this process by delivering protein substrates in need of chaperoning to DnaK and stimulating ATP hydrolysis (58). Of the three J-domain cochaperones in enteric bacteria, DnaJ delivers the largest number of substrates and is thus the major contributor to canonical DnaK-mediated chaperoning, followed by CbpA and DjlA (59). *S*. Typhimurium appears to limit canonical chaperoning by the DnaK/J-domain cochaperone/GrpE system when experiencing cytoplasmic Mg^2+^ starvation because it represses expression of two out of its three J-domain cochaperone-encoding genes in a PhoP-dependent manner (Fig. 2G; Fig. S1B and D), and the substrate pools of J-domain cochaperones share limited overlap that would otherwise raise the possibility of compensation (48, 60).

Classical co-regulation of genes in an operon is facilitated by a promoter that drives co-expression of two or more genes (61). For example, the *groES* and *groEL* genes, which specify the GroES/GroEL chaperone system, are organized in an operon and co-expressed (57). The *groESgroEL* operon has been strictly conserved (43), in contrast to the *dnaK* and *dnaJ*-containing operons, which appear to have undergone evolutionary changes and differ across species. Bacteria vary considerably in the other genes present in the *dnaKdnaJ* operons and the order of the genes, which affects the distance between *dnaK* and *dnaJ* and introduces additional regulatory elements. For example, the *dnaK* and *dnaJ* genes are 1,769 kb apart in the gut commensal bacterium *B. thetaiotaomicron* (Fig. 8A).

During cytoplasmic Mg^2+^ starvation, the *dnaKdnaJ* operon may be transcribed as a single message, as during nutrient abundance, followed by cleavage into two or more mRNAs that differ in stability. In agreement with this notion, PhoP decreased the stability of the *dnaJ* portion of the mRNA but not of that corresponding to *dnaK* (Fig. 5D and E). Alternatively, transcription may terminate in the intergenic region between *dnaK* and *dnaJ*, as previously observed in some bacterial transcripts (62, 63); however, sequences resembling an intrinsic transcription terminator are not present in the *dnaK-dnaJ* intergenic region, which, at 85 nt (Fig. 5A), is unusually long for genes that are part of an operon. A long intergenic region separates the *dnaK* and *dnaJ* genes in other species as well. For example, it is 301 nt long in the tropical spirochete *Leptospira interrogans* (Fig. 8D), reinforcing the notion that *dnaK* and *dnaJ* are differentially expressed under some conditions in different bacterial species. The *dnaKdnaJ* intergenic region may be targeted by a small noncoding RNA (sRNA) that regulates stability of the bicistronic transcript: in both *E. coli* and *S*. Typhimurium, the intergenic region encodes an sRNA, *tpke11*, that shares 80% identity between the two species (64, 65). Expression of *tpke11* is downregulated in a PhoP-dependent manner when *S*. Typhimurium infects rat fibroblasts (65), raising the possibility that bacteria control differential versus coordinate regulation of *dnaK* and *dnaJ* under PhoP-inducing stress conditions (e.g., Mg^2+^ starvation) via *tpke11*.

During nutrient abundance, co-regulation of the *dnaK* and *dnaJ* genes facilitates stoichiometric balance of their encoded proteins (66), which is important for the canonical activities of the DnaK/DnaJ/GrpE chaperone system (67). The differential expression of the *dnaK* and *dnaJ* genes taking place during cytoplasmic Mg^2+^ starvation results in a higher DnaK to DnaJ ratio than during unstressed growth (Fig. 3A). Accordingly, increased free DnaK can potentially interact with cellular structures other than J-domain cochaperones and adopt other functions, such as binding ribosomes and repressing translation (8), which promotes survival against low cytoplasmic Mg^2+^ (8).

RpoH activates transcription from both the *dnaKdnaJ* and *groESgroEL* promoters (5). Cytoplasmic Mg^2+^ starvation promotes transcription of *dnaK* (Fig. 2A) by decreasing the concentration of adenosine triphosphate (ATP) below the threshold necessary for full proteolysis of regulatory proteins (Fig. 1E), including RpoH (36) in a *phoP*-dependent manner (Fig. 6B). The resulting *phoP-*dependent accumulation of RpoH during cytoplasmic Mg^2+^ starvation (Fig. 6C) is reminiscent of that taking place during heat stress (5), albeit caused by a different mechanism: DnaK becomes unavailable to deliver RpoH to ATP-dependent proteases when misfolded proteins titrate DnaK (Fig. 1D) (19, 68).

Finally, DnaK exerts negative feedback on RpoH amounts (Fig. 7A) and activity via its C-terminal 74 residues (Fig. 7B and C). This feedback takes place both when bacteria face cytoplasmic Mg^2+^ starvation (Fig. 7B) and Mg^2+^-abundant conditions (Fig. 7C). Most significantly, DnaK downregulates RpoH in *S*. Typhimurium even in the absence of J-domain cochaperones (Fig. 7), suggesting that this control does not operate via the canonical DnaK pathway involving DnaJ and GrpE. Cumulatively, our findings indicate that the specific stress perturbing protein homeostasis determines the distinct versus coordinate expression and activity of DnaK and its cochaperones.

## MATERIALS AND METHODS

### Bacterial strains and growth conditions

Bacterial strains and oligonucleotides used in this study are presented in Tables S1 and S2. *S.* Typhimurium strains are derived from wild-type strain 14028s.

Typhimurium strains were grown in N-minimal media (pH 7.7) supplemented with 0.1% casamino acids, 38 mM glycerol, and the indicated concentrations of MgCl_2_ at 37°C in a water bath with 250 rpm shaking. Overnight cultures were grown in media with high (10 mM) Mg^2+^, washed three times in media with no Mg^2+^, and diluted 1:50 into media with the indicated concentrations of Mg^2+^. Growth times in low versus high Mg^2+^ (5 h and 4.5 h, respectively) yielded cultures at similar optical densities at 600 nm.

For strains harboring the AtpAGD-expressing plasmid, cells were washed three times with no Mg²^+^ media and then diluted 1:25 into low Mg²^+^ (10 µM) media containing 50 µg/mL ampicillin for plasmid maintenance. Expression of the soluble subunit of the F₁F₀ ATP synthase (AtpAGD) was induced with 1 mM of isopropyl β-D-1-thiogalactopyranoside (IPTG) after 2.5 h of growth.

### RNA extraction and quantitative real-time PCR

Bacterial cultures were grown in N-minimal media as described above. At the indicated times, 0.5 mL of bacterial culture was combined with 1 mL of RNAprotect Bacteria Reagent (Qiagen) to stabilize RNAs. Cells were collected by centrifugation for 10 min (20,000 × *g*, 4°C).

Cell pellets were resuspended in 100 µL Tris-EDTA (TE) with 2 mg/mL lysozyme to lyse bacteria. Total RNA extraction was performed using the RNeasy Kit (Qiagen). A total of 1 µg of RNA was used to synthesize cDNA using SuperScript VILO Master Mix (Thermo Fisher Scientific). The resulting cDNA was diluted 1:10 (for the *dnaK*, *dnaJ*, *cbpA*, *djlA*, and *mgtB* genes) or 1:100 (for the *ompA* and *ffh* genes, used as constitutive controls) for qRT-PCR, which was performed using Fast SYBR Green Master Mix (Thermo Fisher Scientific) using 2 µL of the diluted cDNA. Relative amounts of mRNA corresponding to each gene were determined using a standard curve of *S*. Typhimurium genomic DNA serially diluted by factors of 10.

### *In vivo* RNA stability assay

Bacterial cultures were grown in 10 mL N-minimal media as described above. After 5 h of growth, a pre-rifampicin aliquot of 0.5 mL was collected and combined with RNAprotect Bacteria Reagent (Qiagen) as described above. Rifampicin was added at a final concentration of 200 µg/mL to halt RNA synthesis. Subsequent 0.5 mL samples were collected at the indicated time points.

RNA extraction and qRT-PCR were performed as described above. mRNA values at indicated time points were divided by the initial mRNA value prior to rifampicin addition to determine the fraction of remaining mRNA.

### Western blot

Bacterial cultures were grown in N-minimal media as described above. At the indicated times, the optical density at 600 nm was measured. An equivalent number of cells (OD 0.5 = 0.5 mL) was harvested for each culture and pelleted by centrifugation for 2 min (20,000 × *g*, 4°C). The supernatant was removed, and pellets were resuspended in 50 µL of B-PER Bacterial Protein Extraction Reagent (Thermo Fisher Scientific) supplemented with 100 µg/mL lysozyme. Following 5 min of lysis at room temperature, 50 µL of 2× Laemmli sample buffer was added, and the samples were boiled at 95°C for 10 min. A 5 µL portion of the mixture was electrophoresed on NuPAGE 4%–12% Bis-Tris gels (Thermo Fisher Scientific) and transferred to nitrocellulose membranes using the iBlot 2 transfer device (Thermo Fisher Scientific). Following transfer, membranes were blocked in 3% skim milk in Tris-buffered saline with 0.1% Tween-20 (TBST) at room temperature for 2 h, incubated with the indicated primary antibodies in TBST for 1 h, washed three times, incubated with fluorescent secondary antibodies in TBST for 1 h, and washed three times. Rabbit anti-DnaK (Thermo Fisher Scientific) was used at a dilution of 1:5,000. Rabbit anti-DnaJ (gift from Pierre Genevaux) was used at a dilution of 1:2,500. IRDye 800CW donkey anti-rabbit (LI-COR Biosciences) was used at a 1:5,000 dilution to probe for DnaK and 1:500 dilution to probe for DnaJ. The fluorescent signal was captured with an Amersham ImageQuant 800 imager (Cytiva Life Sciences) using a 775 nm excitation wavelength and IRlong emission filter.

For detection of RpoH, a chemiluminescent method was used. Membranes were blocked as described above, then incubated overnight at 4°C with mouse anti-RpoH antibody (3RH3, BioLegend) diluted 1:2,000 in TBST. Membranes were washed three times in TBST, then incubated for 1 h at room temperature with anti-mouse IgG HRP-conjugated secondary antibody (Promega) diluted 1:5,000 in TBST. Blots were developed using the SuperSignal West Femto substrate (Thermo Fisher Scientific).

### ATP measurement in bacterial cells

Bacterial cultures were grown for the indicated times as described above. ATP measurements were performed using the BacTiter-Glo Microbial Cell Viability Assay Kit (Promega) following the manufacturer’s instructions. ATP levels were normalized to the optical density of the cultures at 600 nm.

## SUPPLEMENTAL MATERIAL

Supplemental material mbio.00227-25-s0001.pdf Fig. S1 to S4 and Tables S1 and S2.

ASM does not own the copyrights to Supplemental Material that may be linked to, or accessed through, an article. The authors have granted ASM a non-exclusive, world-wide license to publish the Supplemental Material files. Please contact the corresponding author directly for reuse.

## References

[1] Mogk A, Huber D, Bukau B. 2011. Integrating protein homeostasis strategies in prokaryotes. Cold Spring Harb Perspect Biol 3:a004366. doi:10.1101/cshperspect.a004366 21441580 PMC3062218

[2] Netzer WJ, Hartl FU. 1998. Protein folding in the cytosol: chaperonin-dependent and -independent mechanisms. Trends Biochem Sci 23:68–73. doi:10.1016/s0968-0004(97)01171-7 9538692

[3] Kerner MJ, Naylor DJ, Ishihama Y, Maier T, Chang H-C, Stines AP, Georgopoulos C, Frishman D, Hayer-Hartl M, Mann M, Hartl FU. 2005. Proteome-wide analysis of chaperonin-dependent protein folding in Escherichia coli. Cell 122:209–220. doi:10.1016/j.cell.2005.05.028 16051146

[4] Fink AL. 1999. Chaperone-mediated protein folding. Physiol Rev 79:425–449. doi:10.1152/physrev.1999.79.2.425 10221986

[5] Roncarati D, Scarlato V. 2017. Regulation of heat-shock genes in bacteria: from signal sensing to gene expression output. FEMS Microbiol Rev 41:549–574. doi:10.1093/femsre/fux015 28402413

[6] Reichmann D, Voth W, Jakob U. 2018. Maintaining a healthy proteome during oxidative stress. Mol Cell 69:203–213. doi:10.1016/j.molcel.2017.12.021 29351842 PMC5777170

[7] Koubek J, Schmitt J, Galmozzi CV, Kramer G. 2021. Mechanisms of cotranslational protein maturation in bacteria. Front Mol Biosci 8:689755. doi:10.3389/fmolb.2021.689755 34113653 PMC8185961

[8] Chan C, Groisman EA. 2024. Chaperone Hsp70 helps Salmonella survive infection-relevant stress by reducing protein synthesis. PLoS Biol 22:e3002560. doi:10.1371/journal.pbio.3002560 38574172 PMC10994381

[9] Hoffmann A, Bukau B, Kramer G. 2010. Structure and function of the molecular chaperone trigger factor. Biochim Biophys Acta 1803:650–661. doi:10.1016/j.bbamcr.2010.01.017 20132842

[10] Lipinska B, King J, Ang D, Georgopoulos C. 1988. Sequence analysis and transcriptional regulation of the Escherichia coil grpE gene, encoding a heat shock protein. Nucleic Acids Res 16:7545–7562. doi:10.1093/nar/16.15.7545 3045760 PMC338426

[11] Bonomo J, Welsh JP, Manthiram K, Swartz JR. 2010. Comparing the functional properties of the Hsp70 chaperones, DnaK and BiP. Biophys Chem 149:58–66. doi:10.1016/j.bpc.2010.04.001 20435400 PMC3175487

[12] Agashe VR, Guha S, Chang H-C, Genevaux P, Hayer-Hartl M, Stemp M, Georgopoulos C, Hartl FU, Barral JM. 2004. Function of trigger factor and DnaK in multidomain protein folding: increase in yield at the expense of folding speed. Cell 117:199–209. doi:10.1016/s0092-8674(04)00299-5 15084258

[13] Calloni G, Chen T, Schermann SM, Chang H-C, Genevaux P, Agostini F, Tartaglia GG, Hayer-Hartl M, Hartl FU. 2012. DnaK functions as a central hub in the E. coli chaperone network. Cell Rep 1:251–264. doi:10.1016/j.celrep.2011.12.007 22832197

[14] Frydman J. 2001. Folding of newly translated proteins In vivo: the role of molecular chaperones. Annu Rev Biochem 70:603–647. doi:10.1146/annurev.biochem.70.1.603 11395418

[15] Tomoyasu T, Mogk A, Langen H, Goloubinoff P, Bukau B. 2001. Genetic dissection of the roles of chaperones and proteases in protein folding and degradation in the Escherichia coli cytosol . Mol Microbiol 40:397–413. doi:10.1046/j.1365-2958.2001.02383.x 11309122

[16] Tilly K, Erickson J, Sharma S, Georgopoulos C. 1986. Heat shock regulatory gene rpoH mRNA level increases after heat shock in Escherichia coli. J Bacteriol 168:1155–1158. doi:10.1128/jb.168.3.1155-1158.1986 2430947 PMC213616

[17] Grossman AD, Straus DB, Walter WA, Gross CA. 1987. Sigma 32 synthesis can regulate the synthesis of heat shock proteins in Escherichia coli. Genes Dev 1:179–184. doi:10.1101/gad.1.2.179 3315848

[18] Miwa T, Taguchi H. 2023. Escherichia coli small heat shock protein IbpA plays a role in regulating the heat shock response by controlling the translation of σ^32^ Proc Natl Acad Sci USA 120:e2304841120. doi:10.1073/pnas.2304841120 37523569 PMC10410725

[19] Straus D, Walter W, Gross CA. 1990. DnaK, DnaJ, and GrpE heat shock proteins negatively regulate heat shock gene expression by controlling the synthesis and stability of sigma 32. Genes Dev 4:2202–2209. doi:10.1101/gad.4.12a.2202 2269429

[20] Tatsuta T, Tomoyasu T, Bukau B, Kitagawa M, Mori H, Karata K, Ogura T. 1998. Heat shock regulation in the ftsH null mutant of Escherichia coli: dissection of stability and activity control mechanisms of sigma32 in vivo. Mol Microbiol 30:583–593. doi:10.1046/j.1365-2958.1998.01091.x 9822823

[21] Arsène F, Tomoyasu T, Bukau B. 2000. The heat shock response of Escherichia coli. Int J Food Microbiol 55:3–9. doi:10.1016/s0168-1605(00)00206-3 10791710

[22] Zhao K, Liu M, Burgess RR. 2005. The global transcriptional response of Escherichia coli to induced sigma 32 protein involves sigma 32 regulon activation followed by inactivation and degradation of sigma 32 in vivo. J Biol Chem 280:17758–17768. doi:10.1074/jbc.M500393200 15757896

[23] Straus DB, Walter WA, Gross CA. 1989. The activity of sigma 32 is reduced under conditions of excess heat shock protein production in Escherichia coli*.* Genes Dev 3:2003–2010. doi:10.1101/gad.3.12a.2003 2695391

[24] Tilly K, McKittrick N, Zylicz M, Georgopoulos C. 1983. The dnaK protein modulates the heat-shock response of Escherichia coli. Cell 34:641–646. doi:10.1016/0092-8674(83)90396-3 6311435

[25] McCarty JS, Rüdiger S, Schönfeld HJ, Schneider-Mergener J, Nakahigashi K, Yura T, Bukau B. 1996. Regulatory region C of the E. coli heat shock transcription factor, sigma32, constitutes a DnaK binding site and is conserved among eubacteria. J Mol Biol 256:829–837. doi:10.1006/jmbi.1996.0129 8601834

[26] Morita MT, Kanemori M, Yanagi H, Yura T. 2000. Dynamic interplay between antagonistic pathways controlling the sigma 32 level in Escherichia coli. Proc Natl Acad Sci USA 97:5860–5865. doi:10.1073/pnas.080495197 10801971 PMC18524

[27] Bahl H, Echols H, Straus DB, Court D, Crowl R, Georgopoulos CP. 1987. Induction of the heat shock response of E. coli through stabilization of sigma 32 by the phage lambda cIII protein. Genes Dev 1:57–64. doi:10.1101/gad.1.1.57 2962898

[28] Cunrath O, Bumann D. 2019. Host resistance factor SLC11A1 restricts Salmonella growth through magnesium deprivation. Science 366:995–999. doi:10.1126/science.aax7898 31753999

[29] Groisman EA, Chan C. 2021. Cellular adaptations to cytoplasmic Mg(2+) limitation. Annu Rev Microbiol 75:649–672. doi:10.1146/annurev-micro-020518-115606 34623895

[30] Bruna RE, Kendra CG, Groisman EA, Pontes MH. 2021. Limitation of phosphate assimilation maintains cytoplasmic magnesium homeostasis. Proc Natl Acad Sci USA 118:e2021370118. doi:10.1073/pnas.2021370118 33707210 PMC7980370

[31] Lee EJ, Pontes MH, Groisman EA. 2013. A bacterial virulence protein promotes pathogenicity by inhibiting the bacterium’s own F1Fo ATP synthase. Cell 154:146–156. doi:10.1016/j.cell.2013.06.004 23827679 PMC3736803

[32] Kleczkowski LA, Igamberdiev AU. 2021. Magnesium signaling in plants. Int J Mol Sci 22:1159. doi:10.3390/ijms22031159 33503839 PMC7865908

[33] Kleczkowski LA, Igamberdiev AU. 2023. Magnesium and cell energetics: at the junction of metabolism of adenylate and non-adenylate nucleotides. J Plant Physiol 280:153901. doi:10.1016/j.jplph.2022.153901 36549033

[34] Patel A, Malinovska L, Saha S, Wang J, Alberti S, Krishnan Y, Hyman AA. 2017. ATP as a biological hydrotrope. Science 356:753–756. doi:10.1126/science.aaf6846 28522535

[35] Pontes MH, Yeom J, Groisman EA. 2016. Reducing ribosome biosynthesis promotes translation during Low Mg2+ Stress. Mol Cell 64:480–492. doi:10.1016/j.molcel.2016.05.008 27746019 PMC5500012

[36] Yeom J, Groisman EA. 2021. Reduced ATP-dependent proteolysis of functional proteins during nutrient limitation speeds the return of microbes to a growth state. Sci Signal 14:eabc4235. doi:10.1126/scisignal.abc4235 33500334 PMC8378506

[37] Herman C, Thévenet D, D’Ari R, Bouloc P. 1995. Degradation of sigma 32, the heat shock regulator in Escherichia coli, is governed by HflB. Proc Natl Acad Sci USA 92:3516–3520. doi:10.1073/pnas.92.8.3516 7724592 PMC42198

[38] Kanemori M, Nishihara K, Yanagi H, Yura T. 1997. Synergistic roles of HslVU and other ATP-dependent proteases in controlling in vivo turnover of sigma32 and abnormal proteins in Escherichia coli. J Bacteriol 179:7219–7225. doi:10.1128/jb.179.23.7219-7225.1997 9393683 PMC179669

[39] Tomoyasu T, Gamer J, Bukau B, Kanemori M, Mori H, Rutman AJ, Oppenheim AB, Yura T, Yamanaka K, Niki H. 1995. Escherichia coli FtsH is a membrane-bound, ATP-dependent protease which degrades the heat-shock transcription factor sigma 32. EMBO J 14:2551–2560. doi:10.1002/j.1460-2075.1995.tb07253.x 7781608 PMC398369

[40] Park SY, Groisman EA. 2014. Signal-specific temporal response by the Salmonella PhoP/PhoQ regulatory system. Mol Microbiol 91:135–144. doi:10.1111/mmi.12449 24256574 PMC3890429

[41] Yeom J, Wayne KJ, Groisman EA. 2017. Sequestration from protease adaptor confers differential stability to protease substrate. Mol Cell 66:234–246. doi:10.1016/j.molcel.2017.03.009 28431231 PMC5424706

[42] Groisman EA, Duprey A, Choi J. 2021. How the PhoP/PhoQ system controls virulence and Mg(2+) homeostasis: lessons in signal transduction, pathogenesis, physiology, and evolution. Microbiol Mol Biol Rev 85:e0017620. doi:10.1128/MMBR.00176-20 34191587 PMC8483708

[43] Segal R, Ron EZ. 1996. Regulation and organization of the groE and dnaK operons in Eubacteria. FEMS Microbiol Lett 138:1–10. doi:10.1111/j.1574-6968.1996.tb08126.x 8674965

[44] Moreno-Hagelsieb G, Collado-Vides J. 2002. A powerful non-homology method for the prediction of operons in prokaryotes. Bioinformatics 18(Suppl 1):S329–S336. doi:10.1093/bioinformatics/18.suppl_1.s329 12169563

[45] Cromie MJ, Shi Y, Latifi T, Groisman EA. 2006. An RNA sensor for intracellular Mg(2+). Cell 125:71–84. doi:10.1016/j.cell.2006.01.043 16615891

[46] Groisman EA, Chan C. 2021. Cellular adaptations to cytoplasmic Mg2+ Lim^it^ation. Annu Rev Microbiol 75:649–672. doi:10.1146/annurev-micro-020518-115606 34623895

[47] Yeom J, Pontes MH, Choi J, Groisman EA. 2018. A protein that controls the onset of a Salmonella virulence program. EMBO J 37:e96977. doi:10.15252/embj.201796977 29858228 PMC6043847

[48] Gur E, Biran D, Shechter N, Genevaux P, Georgopoulos C, Ron EZ. 2004. The Escherichia coli DjlA and CbpA proteins can substitute for DnaJ in DnaK-mediated protein disaggregation. J Bacteriol 186:7236–7242. doi:10.1128/JB.186.21.7236-7242.2004 15489435 PMC523209

[49] Zwir I, Latifi T, Perez JC, Huang H, Groisman EA. 2012. The promoter architectural landscape of the Salmonella PhoP regulon. Mol Microbiol 84:463–485. doi:10.1111/j.1365-2958.2012.08036.x 22435712 PMC3335776

[50] Cowing DW, Bardwell JC, Craig EA, Woolford C, Hendrix RW, Gross CA. 1985. Consensus sequence for Escherichia coli heat shock gene promoters. Proc Natl Acad Sci USA 82:2679–2683. doi:10.1073/pnas.82.9.2679 3887408 PMC397628

[51] Koebmann BJ, Westerhoff HV, Snoep JL, Nilsson D, Jensen PR. 2002. The glycolytic flux in Escherichia coli is controlled by the demand for ATP. J Bacteriol 184:3909–3916. doi:10.1128/JB.184.14.3909-3916.2002 12081962 PMC135175

[52] Craig EA, Gross CA. 1991. Is hsp70 the cellular thermometer? Trends Biochem Sci 16:135–140. doi:10.1016/0968-0004(91)90055-z 1877088

[53] Bukau B. 1993. Regulation of the Escherichia coli heat-shock response. Mol Microbiol 9:671–680. doi:10.1111/j.1365-2958.1993.tb01727.x 7901731

[54] Juhas M, van der Meer JR, Gaillard M, Harding RM, Hood DW, Crook DW. 2009. Genomic islands: tools of bacterial horizontal gene transfer and evolution. FEMS Microbiol Rev 33:376–393. doi:10.1111/j.1574-6976.2008.00136.x 19178566 PMC2704930

[55] Lawrence JG. 1997. Selfish operons and speciation by gene transfer. Trends Microbiol 5:355–359. doi:10.1016/S0966-842X(97)01110-4 9294891

[56] Mayer MP, Bukau B. 2005. Hsp70 chaperones: cellular functions and molecular mechanism. Cell Mol Life Sci 62:670–684. doi:10.1007/s00018-004-4464-6 15770419 PMC2773841

[57] Kröger C, Colgan A, Srikumar S, Händler K, Sivasankaran SK, Hammarlöf DL, Canals R, Grissom JE, Conway T, Hokamp K, Hinton JCD. 2013. An infection-relevant transcriptomic compendium for salmonella enterica serovar typhimurium. Cell Host Microbe 14:683–695. doi:10.1016/j.chom.2013.11.010 24331466

[58] Liberek K, Marszalek J, Ang D, Georgopoulos C, Zylicz M. 1991. Escherichia coli DnaJ and GrpE heat shock proteins jointly stimulate ATPase activity of DnaK. Proc Natl Acad Sci USA 88:2874–2878. doi:10.1073/pnas.88.7.2874 1826368 PMC51342

[59] Sugimoto S, Yamanaka K, Niwa T, Terasawa Y, Kinjo Y, Mizunoe Y, Ogura T. 2021. Hierarchical model for the role of J-domain proteins in distinct cellular functions. J Mol Biol 433:166750. doi:10.1016/j.jmb.2020.166750 33310019

[60] Chae C, Sharma S, Hoskins JR, Wickner S. 2004. CbpA, a DnaJ homolog, is a DnaK Co-chaperone, and its activity Is modulated by CbpM. J Biol Chem 279:33147–33153. doi:10.1074/jbc.M404862200 15184371

[61] Sadava DE. 2011. Life: the science of biology. W. H. Freeman.

[62] Nickel CM, Vandekerckhove J, Beyer P, Tadros MH. 1997. Molecular analysis of the Rhodobacter capsulatus chaperone dnaKJ operon: purification and characterization of DnaK. Gene 192:251–259. doi:10.1016/s0378-1119(97)00085-1 9224898

[63] Segal G, Ron EZ. 1995. The dnaKJ operon of Agrobacterium tumefaciens: transcriptional analysis and evidence for a new heat shock promoter. J Bacteriol 177:5952–5958. doi:10.1128/jb.177.20.5952-5958.1995 7592349 PMC177424

[64] Rivas E, Klein RJ, Jones TA, Eddy SR. 2001. Computational identification of noncoding RNAs in E. coli by comparative genomics. Curr Biol 11:1369–1373. doi:10.1016/S0960-9822(01)00401-8 11553332

[65] Ortega AD, Gonzalo-Asensio J, García-del Portillo F. 2012. Dynamics of Salmonella small RNA expression in non-growing bacteria located inside eukaryotic cells. RNA Biol 9:469–488. doi:10.4161/rna.19317 22336761

[66] Rocha EPC. 2008. The organization of the bacterial genome. Annu Rev Genet 42:211–233. doi:10.1146/annurev.genet.42.110807.091653 18605898

[67] Srinivasan SR, Gillies AT, Chang L, Thompson AD, Gestwicki JE. 2012. Molecular chaperones DnaK and DnaJ share predicted binding sites on most proteins in the E. coli proteome. Mol Biosyst 8:2323–2333. doi:10.1039/c2mb25145k 22732719 PMC3462289

[68] Tomoyasu T, Ogura T, Tatsuta T, Bukau B. 1998. Levels of DnaK and DnaJ provide tight control of heat shock gene expression and protein repair in Escherichia coli . Mol Microbiol 30:567–581. doi:10.1046/j.1365-2958.1998.01090.x 9822822

