# Supplementary material for: Infection-relevant conditions dictate differential versus coordinate expression of Salmonella chaperones and cochaperones
Source: mBio. 2025 Mar 31;16(5):e00227-25. doi: 10.1128/mbio.00227-25 (PMC12077118; doi:10.1128/mbio.00227-25)
Supplement: Supplemental material — Fig. S1 to S4 and Tables S1 and S2. [file mbio.00227-25-s0001.pdf]

Supplemental Material for

Infection-relevant conditions dictate differential versus coordinate expression of *Salmonella* chaperones and cochaperones

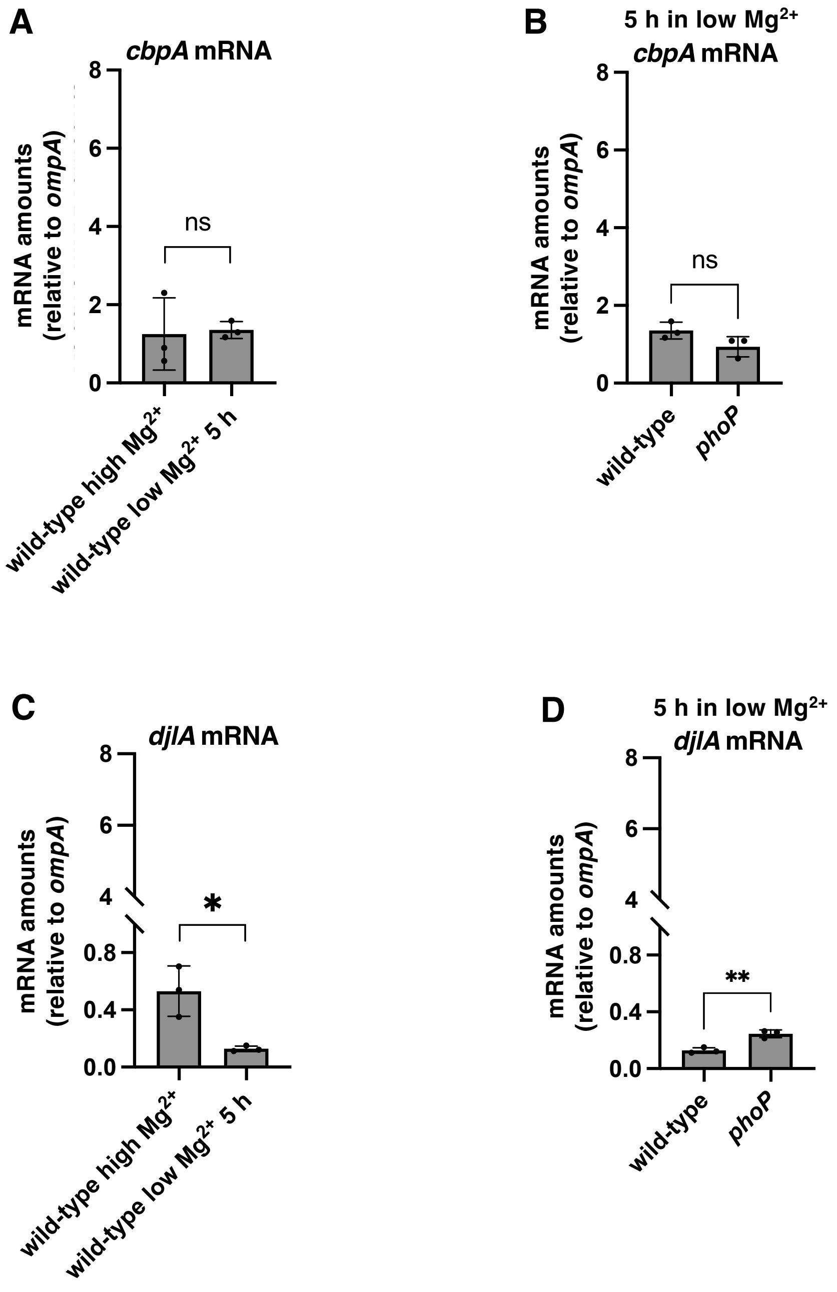
A
cbpA mRNA
mRNA amounts (relative to ompA)
8
6
4
2
0
ns
wild-type high Mg2+
wild-type low Mg2+ 5 h
B
5 h in low Mg2+
cbpA mRNA
mRNA amounts (relative to ompA)
ns
wild-type
phoP
C
djlA mRNA
mRNA amounts (relative to ompA)
0.8
0.4
0.0
*
wild-type high Mg2+
wild-type low Mg2+ 5 h
D
5 h in low Mg2+
djlA mRNA
mRNA amounts (relative to ompA)
**
wild-type
phoP

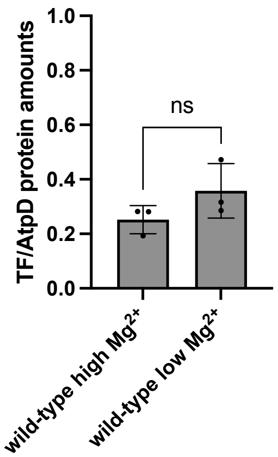

TF/AtpD protein amounts
1.0
0.8
0.6
0.4
0.2
0.0
ns
wild-type high Mg2+
wild-type low Mg2+

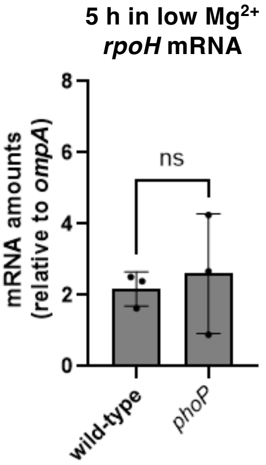

5 h in low Mg2+
rpoH mRNA
mRNA amounts
(relative to ompA)
8
6
4
2
0
ns
wild-type
phoP

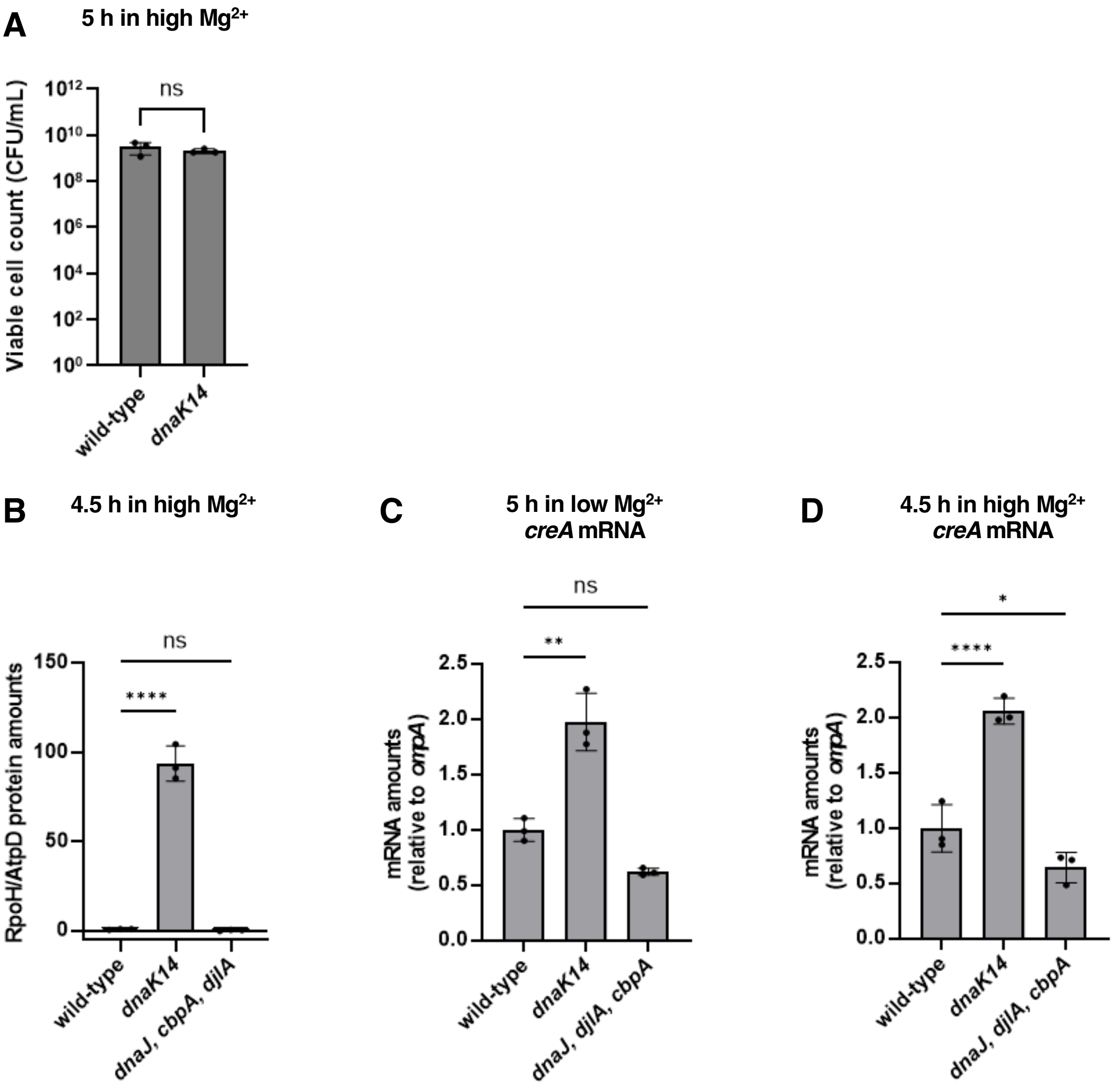
A
5 h in high Mg2+
Viable cell count (CFU/mL)
ns
wild-type
dnaK14
B
4.5 h in high Mg2+
RpoH/AtpD protein amounts
ns
****
wild-type
dnaK14
dnaJ, cbpA, djlA
C
5 h in low Mg2+
creA mRNA
mRNA amounts (relative to ompA)
ns
**
wild-type
dnaK14
dnaJ, djlA, cbpA
D
4.5 h in high Mg2+
creA mRNA
mRNA amounts (relative to ompA)
*
****
wild-type
dnaK14
dnaJ, djlA, cbpA

Supporting Figure Legends

Supporting Figure S1: The mRNA abundance of J-protein cochaperone-encoding genes does not increase when *S.* Typhimurium experiences cytoplasmic Mg^{2+} starvation.

(A) mRNA abundance of the *cbpA* gene relative to the constitutive *ompA* control in wild-type (14028s) *S.* Typhimurium following 4.5 h of growth in high (10 mM) Mg^{2+} or 5 h of growth in low (10 μM) Mg^{2+}.

(B) mRNA abundance of the *cbpA* gene relative to the constitutive *ompA* control in wild-type (14028s) and *phoP* (MS7953s) *S.* Typhimurium following 5 h of growth in low (10 μM) Mg^{2+}.

(C) mRNA abundance of the *djlA* gene relative to the constitutive *ompA* control in wild-type (14028s) *S.* Typhimurium following 4.5 h of growth in high (10 mM) Mg^{2+} or 5 h of growth in low (10 μM) Mg^{2+}.

(D) mRNA abundance of the *djlA* gene relative to the constitutive *ompA* control in wild-type (14028s) and *phoP* (MS7953s) *S.* Typhimurium following 5 h of growth in low (10 μM) Mg^{2+}.

Data in (A-D) represent mean ± SD of three independent biological replicates. Statistical analysis was performed using two-tailed Student's *t*-test comparing the bracketed sample groups (*$p<0.05$, **$p<0.01$, ns = not significant).

Supporting Figure S2: Trigger Factor protein amounts are similar during growth in high Mg^{2+} and low Mg^{2+}.

Protein amounts of TF relative to the AtpD loading control determined by Western blot in wild-type (14028s) *S.* Typhimurium following 4.5 h of growth in high (10 mM) Mg^{2+} or 5 h of growth in low (10 μM) Mg^{2+}.

Data represent mean ± SD of three independent biological replicates. Statistical analysis was performed using two-tailed Student's *t*-test comparing the bracketed sample groups (ns = not significant).

Supporting Figure S3: Inactivation of the *phoP* gene does not alter *rpoH* mRNA amounts.

mRNA abundance of the *rpoH* gene relative to the constitutive *ompA* control in wild-type (14028s) and *phoP* (MS7953s) *S.* Typhimurium following 5 h of growth in low (10 μM) Mg^{2+}.

Data represent mean ± SD of three independent biological replicates. Statistical analysis was performed using two-tailed Student's *t*-test comparing the bracketed sample groups (ns = not significant).

Supporting Figure 4: C-terminal domain of DnaK is required to decrease RpoH amounts and activity during growth in high Mg^{2+}.

(A) Survival of wild-type (14028s) and *dnaK14* (CC186) *S.* Typhimurium following 5 h of growth in low (10 μM) Mg^{2+}.

(B) Protein amounts of RpoH relative to the AtpD loading control determined by Western blot in wild-type (14028s), *dnaK14* (CC186), and *dnaJ cbpA djlA* (CC656) *S.* Typhimurium following 4.5 h of growth in high (10 mM) Mg^{2+}.

(**C**) mRNA abundance of the *creA* gene relative to that of the constitutive *ompA* control in wild-type (14028s), *dnaK14* (CC186), and *dnaJ cbpA djlA* (CC656) *S.* Typhimurium following 5 h of growth in low (10 μM) Mg^{2+}.

(**D**) mRNA abundance of the *creA* gene relative to that of the constitutive *ompA* control in wild-type (14028s), *dnaK14* (CC186), and *dnaJ cbpA djlA* (CC656) *S.* Typhimurium following 4.5 h of growth in high (10 mM) Mg^{2+}.

Data in **(A-C)** represent mean ± SD of three independent biological replicates. Statistical analysis was performed using two-tailed Student's *t*-test comparing the bracketed sample groups (*p<0.05, **p<0.01, ****p<0.0001, ns = not significant).

Table S1. Strains and plasmids used in this study

Strains	Relevant characteristics	Source
***Salmonella enterica* serovar Typhimurium**		
14028s	wild-type	(Fields et al., 1986)
MS7953s	*phoP*::Tn*10*	(Fields et al., 1986)
EL1	*mgtC*::*kan*	(Lee et al., 2013)
CC186	*dnaK*14::Tn*10d*Cm (insertion at nucleotide position 1691)	(Chan and Groisman, 2024)
CC656	*dnaJ, cbpA, djlA*::*kan*	(Chan and Groisman, 2024)
Escherichia coli		
DH5*α*	Host strain used for generation and propagation of plasmid constructs	(Hanahan, 1985)
**Plasmids**		
pUHE-21-2-lacI^q	rep$_{pMB1}$ lacI^q Amp^R vector control	(Soncini et al., 1995)
pAtpAGD	pUHE-21-2-lacI^q-AtpAGD	(Pontes et al., 2016)

Table S2. Oligonucleotides used in this study

Oligo name	Sequence (5′ to 3′)	Purpose	Source
17717	TTA TTG GTA TCG ACC TGG GTA CTA C	*dnaK* mRNA qRT-PCR quantification	This work
17718	CAG AGT TTC ACC ATC CTG GGT ATA A	*dnaK* mRNA qRT-PCR quantification	This work
16274	AAGCCTACGAAGTGCT GACC	*dnaJ* mRNA qRT-PCR quantification	This work
16275	ATTAAAGCCGCCGCCA AATC	*dnaJ* mRNA qRT-PCR quantification	This work
18914	CGCCATTATGGGCGTG AAACC	*cbpA* mRNA qRT-PCR quantification	This work
18915	CGGGTTCTTTGCTGACA TCTGG	*cbpA* mRNA qRT-PCR quantification	This work
18916	gataattggcgtcgccgtagc	*djlA* mRNA qRT-PCR quantification	This work
18917	ccatttttcggctgcgggc	*djlA* mRNA qRT-PCR quantification	This work
15054	gggctggtctcagtaccatga	*ompA* mRNA qRT-PCR quantification	(Yeom et al., 2017)
15055	tcatgagtcgggccatca	*ompA* mRNA qRT-PCR quantification	(Yeom et al., 2017)
19251	gacgacgttgtcgacgctg	*dnaKJ* intergenic region qRT-PCR quantification	This work
19252	ccgctgttttggaaacgcc	*dnaKJ* intergenic region qRT-PCR quantification	This work
19253	ggaaatcgctcagcagcaac	*dnaK* 3' end qRT-PCR quantification	This work
19254	cttcttcaaactcagcgtcgac	*dnaK* 3' end qRT-PCR quantification	This work
19255	ggcgaaaagagattactacgagattt tag	*dnaJ* 5' end qRT-PCR quantification	This work

19256	cggatgatatttcatggccagg	*dnaJ* 5' end qRT-PCR quantification	This work
17697	TAA AAT TCG GTA ACG ACG CTC GTG T	*groEL* mRNA qRT-PCR quantification	This work
17698	ATA GTC GGC GCA CCG AAA GAT TTA T	*groEL* mRNA qRT-PCR quantification	This work
18739	GTA CTT TTC TCA ATT TTG TTG CTG CTG	*creA* mRNA qRT-PCR quantification	This work
18740	CAC CAC GAT TTT ATG GTC AGG ACC	*creA* mRNA qRT-PCR quantification	This work
